# Single TNF*α *trimers mediating NF-*κ*B activation: stochastic robustness of NF-*κ*B signaling

**DOI:** 10.1186/1471-2105-8-376

**Published:** 2007-10-09

**Authors:** Tomasz Lipniacki, Krzysztof Puszynski, Pawel Paszek, Allan R Brasier, Marek Kimmel

**Affiliations:** 1 Institute of Fundamental Technological Research, Swietokrzyska 21, 00-049 Warsaw, Poland; 2 Department of Statistics, Rice University, 6100 Main St. MS-138, Houston, TX 77005, USA; 3 Institute of Automation, Silesian Technical University, 44-100 Gliwice, Poland; 4 Centre for Cell Imaging, School of Biological Sciences, Bioscience Research Building, Crown St., University of Liverpool, Liverpool L69 7ZB, UK; 5 Department of Internal Medicine, University of Texas Medical Branch, Galveston, TX 77555-1060, USA

## Abstract

**Background:**

The NF-*κ*B regulatory network controls innate immune response by transducing variety of pathogen-derived and cytokine stimuli into well defined single-cell gene regulatory events.

**Results:**

We analyze the network by means of the model combining a deterministic description for molecular species with large cellular concentrations with two classes of stochastic switches: cell-surface receptor activation by TNF*α *ligand, and I*κ*B*α *and A20 genes activation by NF-*κ*B molecules. Both stochastic switches are associated with amplification pathways capable of translating single molecular events into tens of thousands of synthesized or degraded proteins. Here, we show that at a low TNF*α *dose only a fraction of cells are activated, but in these activated cells the amplification mechanisms assure that the amplitude of NF-*κ*B nuclear translocation remains above a threshold. Similarly, the lower nuclear NF-*κ*B concentration only reduces the probability of gene activation, but does not reduce gene expression of those responding.

**Conclusion:**

These two effects provide a particular stochastic robustness in cell regulation, allowing cells to respond differently to the same stimuli, but causing their individual responses to be unequivocal. Both effects are likely to be crucial in the early immune response: Diversity in cell responses causes that the tissue defense is harder to overcome by relatively simple programs coded in viruses and other pathogens. The more focused single-cell responses help cells to choose their individual fates such as apoptosis or proliferation. The model supports the hypothesis that binding of single TNF*α *ligands is sufficient to induce massive NF-*κ*B translocation and activation of NF-*κ*B dependent genes.

## Background

Living cells are considered noisy or stochastic biochemical reactors. Most of the cell to cell variability is due to existence of stochastic switches or slow reaction channels involving limited numbers of reacting molecules. Stochastic switches provide inputs for amplification cascades, which translate the single molecule events into a larger population of downstream effector molecules.

The most studied example of stochastic regulation is gene expression, where stochasticity, in eukaryotic organisms, arises mostly from fluctuation in gene activity [1-5] and mRNA synthesis or decay [6-10] reviewed recently in [11]. Control of gene activity is mediated by transcription factors that bind to specific promoter regions, switching the gene *on *or *off*. When the gene is active, RNA polymerase may bind to the gene promoter and enter the transcriptional elongation mode, producing full length pre-mRNA transcripts. The edited mRNA is then exported to the cytoplasm, where the protein translation occurs. In this way, a single gene activation event results (if the activation period is sufficiently long) in a burst of mRNA molecules [12], which is then translated into an even larger burst of proteins.

Another example of stochastic regulation is provided by cell surface receptors and amplification of successive cascade of downstream kinases. There is a large body of evidence that cells are capable of responding to a single- or a very limited number of activating molecules. For example, retinal Rod cells are able to transduce a single photon into a hyperpolarization response [13]. For the present study, signal amplification from a small number of receptors detecting pathogen presence is specially important since it enables cells to respond with protective cytokine cascades to protect the tissue before adaptive immune response can be generated. For example, T-lymphocytes are able to detect a single foreign peptide antigen and only three peptides are required for induction of T-cell cytotoxicity [14,15]. Similar behavior is seen for the toll-interleukin (TIR) superfamily of receptors, where IL-1 signal transduction has been observed in cells expressing only about 10 IL-1 receptors per cell [16]. Similarly, cells respond to TNF*α *stimulation at femtomolar concentration, i.e., when the number of TNF*α *ligands per cell is very limited [17]. In all cases, the activation of receptors leads to the initiation of a signal transduction-amplification cascade, involving activation (phosphorylation) of downstream effector kinases. More generally, it is important to point out that cells can detect and respond to single molecule intracellular events such as DNA damage (leading to p53 activation) or presence of single viral RNAs. Although the phenomenon of cell sensitivity to single activating molecules is well established in biological systems, very few theoretical studies have addressed the effect of stochastic cell surface signaling and its consequences for the downstream cellular responses.

Innate immunity is an intensively studied cellular signaling response to pathogens and pathogen-associated patterns which results in the expression of protective cytokines, such as interferon *β*, that serve to limit the spread of infection until more specific adaptive immunity can be generated. In this regard, the cytoplasmic transcription factor NF-*κ*B is a major mediator of innate immune responses [18,19]. In resting cells NF-*κ*B is sequestered in the cytoplasm by dimerization with inhibitory proteins called I*κ*B. Although several I*κ*B isoforms have been identified, the primary inhibitor is I*κ*B*α*. In the classical NF-*κ*B activation pathway, extracellular signals such as tumor necrosis factor-alpha (TNF*α*) and interleukin-1 (IL-1) bind to cell surface receptors coupled to the cytoplasmic I*κ*B kinase (IKK), a multiprotein complex that phosphorylates I*κ*B*α*, leading to its ubiquitination and then to its rapid proteasomal degradation [20]. Liberated NF-*κ*B is then rapidly translocated into the nucleus to bind to high affinity sites in the genome, thereby influencing target gene expression. Experimental findings have shown that NF-*κ*B nuclear residence is transient and dynamic, an observation that has led to the discovery of negative feedback NF-*κ*B-I*κ*B*α *loop in the NF-*κ*B pathway [21].

The two levels of autoregulatory negative feedback control, termed the NF-*κ*B-I*κ*B*α *and NF-*κ*B-A20-IKK feedback loops, arise because both I*κ*B*α *and A20 genes are directly regulated by NF-*κ*B binding. In the NF-*κ*B-I*κ*B*α *feedback loop, NF-*κ*B enters the nucleus after I*κ*B*α *degradation, NF-*κ*B induces I*κ*B*α *resynthesis to recapture activated nuclear NF-*κ*B and return it to the cytoplasm. In the NF-*κ*B-A20-IKK feedback loop, A20, a protein that is not expressed prior to stimulation is also strongly NF-*κ*B responsive [22]. A20 is an inhibitor of IKK kinase that complexes with an IKK regulatory subunit leading to its inactivation and that induces degradation of RIP a necessary component of the active TNFR1 receptor complex. Without A20 expression, the IKK retains activity which leads to rapid degradation of the newly resynthesized I*κ*B*α*, which destroys the NF-*κ*B-I*κ*B*α *feedback loop [23]. As an illustration, genetically A20 deficient mice are hypersensitive to TNF*α *and develop severe in inflammation even though they have an intact I*κ*B*α *mRNA expression [24].

Nuclear NF-*κ*B activates groups of genes through a process initiated by its binding to high affinity DNA binding sites in regulatory regions of their promoters. Although NF-*κ*B binding to some genes results in rate-limiting complex formation of coactivators, pre-initiation factors, and RNA polymerase (Pol) II, the mode of regulation of rapidly induced negative feedback inhibitors appears to be distinct. Interestingly, chromatin immunoprecipitation assays have shown that the I*κ*B*α *and A20 promoters are "pre-loaded"- already bound by general transcription factors, coactivators and RNA Pol II, waiting for the presence of NF-*κ*B binding to activate expression [25,26]. In these promoters, RNA Pol II is stalled in an activated state; upon NF-*κ*B binding, RNA Pol II enters a competent transcriptional elongation mode and becomes able to transcribe the gene. In this manner, the inhibitory genes of the NF-*κ*B feedback loop are poised to rapidly respond to the presence of nuclear NF-*κ*B.

In last five years several models of the NF-*κ*B signaling regulatory module have been developed, reviewed in [27]. The first attempt was a one-feedback loop model that concentrated on the interplay between the three I*κ*B isoforms [28]. The next attempt was our two feedback loop model [23], incorporating effects of both the I*κ*B*α *and A20 inhibitors. In this model the representation of the NF-*κ*B-I*κ*B regulatory module was simplified by incorporation of only one I*κ*B inhibitor, I*κ*B*α*, that is responsible for the majority of cytoplasmic NF-*κ*B binding and the only one which knockout is lethal, [29]. Incorporating the second NF-*κ*B-A20-IKK negative feedback loop, accurately predicted the time dependent profile of IKK activity. A third model introduced the transduction pathway, from the TNFR1 receptor to activation of the IKKK and IKK kinases and was used to analyze responses of HepG2 cells and HepG2.2.15 (HepG2 cells producing hepatitis B virus) to TNF*α *stimulation [30].

Recently, the NF-*κ*B signaling in single cells was analyzed both experimentally [31-34] and numerically by means of stochastic modeling [35,36], or agent-based modeling [37]. Both analyses indicate that the NF-*κ*B regulatory module demands single cell, stochastic analysis due to cellular heterogeneity and population asynchrony. In the present work we expand our two-feedback loop, single-cell stochastic model of NF-*κ*B activation to incorporate a second stochastic switch at the level of the TNF*α*-TNFR1 interaction. We analyze response of the NF-*κ*B regulatory module over a broad range of stimulation by its activating ligand. We will show, how, although this may seem counter-intuitive, stochasticity and stochastic switches may introduce robustness into the gene regulatory response. In short, the stochastic robustness is due to amplification cascades and progressive signal saturation. As a result, a low amplitude signal (small concentration of activating ligand or transcription factor) leads to an "almost" *Yes *or *No *response, with the probability of *Yes *being a function of the input signal amplitude. This type of regulation enables cells to chose a well-defined fate such as signaling, apoptosis, or others, but in addition it allows individual cell responses to vary. In the range of stimulation amplitudes, for which most cells follow the same evolution path, the cell population-based experiments and modeling are well justified. However, in the case when population is a mixture of differently responding cells (for example apoptotic and proliferating or TNF*α *responding or not) the average trajectory does not represent any biological process and the model reproducing such trajectory is likely to be incorrect.

## Results and discussion

### Stochastic switches and amplification cascades in NF-*κ*B regulation

**Figure 1:**
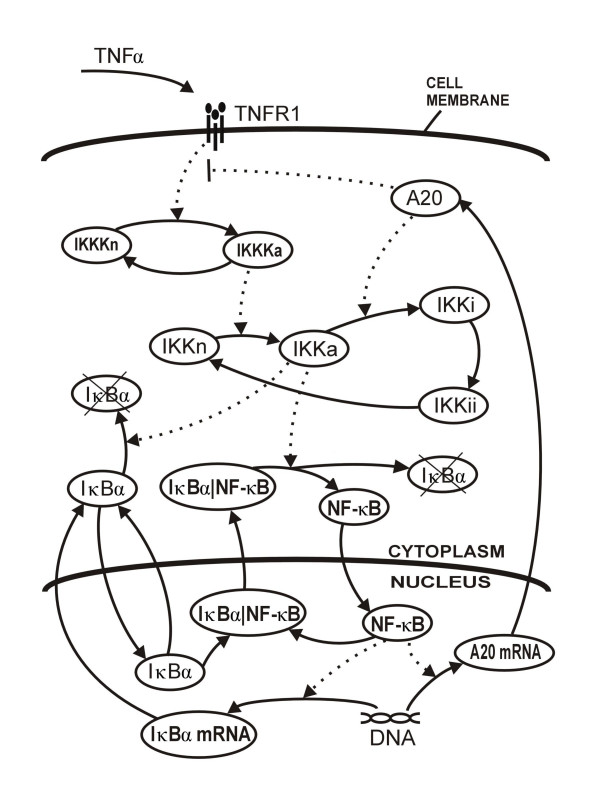
Two feedback-loop model of NF-*κ*B regulatory pathway.

Our considerations are based on the two-feedback loop stochastic model of the NF-*κ*B pathway, which combines the signal transduction cascade that connects cell surface receptors with the core regulatory module analyzed previously [35]. Current model involves two-compartment kinetics of transcription factor NF-*κ*B, its activators, IKKK, IKK and inhibitors, A20 and I*κ*B*α*, shown in Fig. 1. IKKK represents the IKK activating kinase, which itself is activated at the TNFR1 receptor complex (see Materials and Methods for details).

In Additional file 3 (Figs S1, S2, S3 and S4) we demonstrate the model's ability to reproduce major NF-*κ*B pathway experiments on cells exhibiting oscillations in cytoplasmic to nuclear NF-*κ*B localization that arise in response to persistent TNF*α *stimulation.

In the current paper we focus on TNF*α *signaling, a process initiated by binding of TNF*α *to the ubiquitous receptor TNFR1. In short, the action of regulatory pathway may be summarized as follows: Binding of TNF*α *trimer initiates receptor TNFR1 trimerization and formation of an active receptor complex in a multistep process involving binding of RIP and TRAF2. The active receptor complex activates the IKKK kinase (transformation from IKKKn to IKKKa). Active kinase IKKKa phosphorylates and activates the IKK kinase (transformation from IKKn to IKKa). Active IKKa kinase transiently binds to the cytoplasmic (NF-*κ*B|I*κ*B*α*) complex and phosphorylates I*κ*B*α *initiating its degradation. Released NF-*κ*B enters the nucleus to induce transcription of inhibitors I*κ*B*α *and A20 genes. The first negative feedback loop involves the I*κ*B*α *protein, which is rapidly resynthesized, enters the nucleus and recaptures NF-*κ*B back into the cytoplasm. In the continued presence of IKKa, however, the resynthesized I*κ*B*α *would be continuously degraded, which would result in continued nuclear NF-*κ*B translocation. A second level of negative autoregulation occurs with the resynthesis of A20, a ubiquitin ligase which controls IKK activity. A20 initiates degradation of RIP, the key component of TNFR1 receptor complex, what attenuates the activity of receptors and directly associates itself with IKKa, enhancing its conversion to catalytically inactive IKKi. Inactive kinase IKKi spontaneously converts back to IKKn through the intermediate form IKKii. Similarly, active kinase IKKKa rapidly converts to the inactive form IKKKn.

**Figure 2:**
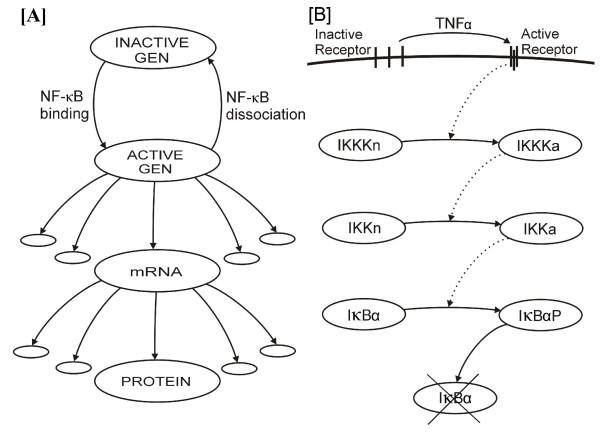
Two ways of signal amplification present in the model. Panel A: A20 (or I*κ*B*α*) gene expression. Panel B: Transduction amplification pathway leading to I*κ*B*α *degradation.

We identified two stochastic processes crucial to the functioning of the NF-*κ*B regulatory pathway: (1) Activation of A20 and I*κ*B*α *genes via binding of NF-*κ*B molecules to the genes promoters and (2) activation of TNFR1 receptors via binding of TNF*α *trimers. These stochastic events may influence the evolution and fate of the cell due to their association with amplification cascades, as shown in Fig. 2 (see [38-40] for discussion of signal and noise propagation in gene neworks and amplification cascades).

Gene expression cascade (Fig. 2A) starts from the activation of a single gene copy, which may then serve as a template for the synthesis of tens or hundreds of mRNA molecules. In turn, a single mRNA molecule is a template for synthesis of hundreds of protein molecules. In this way the two I*κ*B*α *gene copies are sufficient to replenish pool of I*κ*B*α *proteins of about 100,000 molecules, within a half hour. As estimated experimentally by Yang [41] the endogenous level of I*κ*B*α *molecules is 135,000, most of these molecules are degraded at in first 10 min. of high dose TNF*α *stimulation, and then I*κ*B*α *level is approximately restored between 30 and 75 min. of stimulation [28].

At the level of cell surface receptors, a single TNF*α *trimer binding to the TNFR1 receptor leads to receptor trimerization and formation of a stable active receptor complex (Fig 2B). Grell [17] found that TNF*α *trimers dissociate from TNFR1 receptors with a half time of 33 min., while the internalization time is of order of 10 to 20 min. During this time the single active receptor may activate numerous IKKK kinase molecules. In turn, each active IKKKa activates numerous IKK kinases, and each of IKKa may phosphorylate several I*κ*B*α *molecules leading to their degradation. The IKKK-IKK transduction cascade resembles the MAPK cascade and provides signal amplifica-tion of several orders of magnitude [42]. This amplification mechanisms enables cells to respond to femtomolar concentrations of TNF*α *[17,43-45] and references therein.

Recently, in cell population studies, Cheong et al. [45] observed activation of the NF-*κ*B regulatory module in response to TNF*α *concentrations of 0.01 ng/ml. This equals to 200 fM (assuming that TNF*α *consists of trimers of mass 51 kDa) and implies about 1 TNF*α *trimer per 8 × 10^-12^*l *or less than one TNF*α *trimer molecule per volume of mammalian cell which is of the order 2 × 10^-12^*l*. This estimations suggests that cells may be activated by binding of a single, or few, TNF*α *trimers, and that at femtomolar concentration some cells may become active and some not, since TNF*α *binding is a stochastic process. It also indicates that when such small concentrations are considered, the average number of TNF*α *trimers per cell may be a better parameter to describe the experiment than the TNF*α *concentration itself. For example Chan and Aggarwal [44] observed two fold NF-*κ*B induction at TNF*α *dose of 100 fM. For the EMSA assay they incubated 2 × 10^6 ^cells in 500 *μl *medium, which gives 5 TNF trimers per cell. The same concentration in tissue, where the cells are tightly packed would imply less than 1 molecule of activator per cell. The number of TNFR1 receptors per cell may vary significantly between cell lines [44], e.g. there are about 3000 TNFR1 per cell for HeLa [17], and about 10000 for Histiocytic lymphoma (U-937) cells [44], but much less for B-cell lymphoma (Raji) cells. Since in low dose experiments there are more TNFR1 receptors, than TNF*α *molecules, the concentration of free TNF*α *will be influenced by reaction with these receptors and will not remain constant in the course of low dose experiments. Similarly, when the spread of TNF*α *in the tissue is considered one may expect that TNF*α *diffusion will be strongly influenced by binding to free TNFR1 receptors, which may restrict cell to cell signaling to very short distances.

In HL60 cells, NF-*κ*B activity was already observed at TNF*α *concentrations as low as 0.1 pM whereas maximum NF-*κ*B activation required 0.4 to 2 pM TNF*α*, [43].

There is also an ample evidence that cells are able to respond to single viruses, which are known to activate NF-*κ*B pathway through Toll-like receptors dependent and independent pathways, both engaging IKK, [46,47]. Specifically, in a recent analysis of human A549 pulmonary type II epithelial cells infected by respiratory syncytial virus (RSV) at MOI = 1 (multiplicity of infection) we showed that 60% of cell exhibit RelA activation [48]. The MOI = 1 implies (if the Poisson distribution of virions per cell is assumed) that 37% of cell will remain uninfected, while only 26% of cell will be infected by more than 1 virion. Thus the observed 60% fraction of responding cells implies that a single virus is enough to induce NF-*κ*B activity in a cell. Arnold et al. [49] analyzed responses of peripheral blood mononuclear cells (PBMC) after exposure to low infectious RSV doses, with MOI from 0.001 to 1. Even at MOI as low as 0.01–0.1 they observed pronounced secretion of, NF-*κ*B responsive cytokines like IL-8, IL-6 and TNF*α *at 4 hours after infection.

### System recovery: pulse-pulse experiment

**Figure 3:**
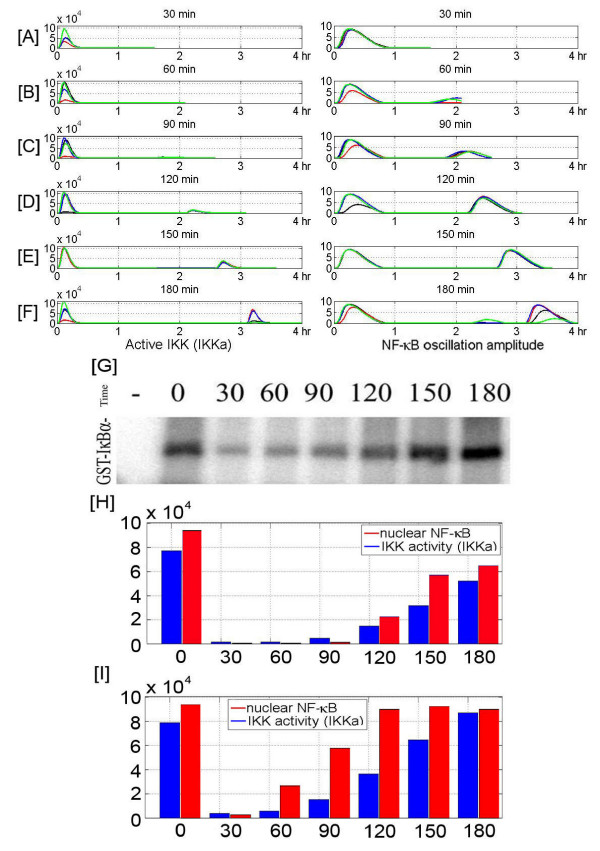
Pulse-Pulse experiment. Panels A to F: IKKa and NF-*κ*B oscillation amplitude resulting from single cell numerical simulations. Two TNF*α *(20 ng/ml), 5 min pulses are separated by 30', 60', 90' 120' 150' or 180' breaks. Four single cell simulations (marked by different colors) are shown in each panel. Panel G: IKK kinase activity in 3T3 cells stimulated by two 5 min. pulses of TNF*α *(20 ng/ml), separated by 30', 60', 90' 120' 150' or 180' break. First sub-blot corresponds to unstimulated cells, second sub-blot shows intensity of first peak, while next six sub-blots show the intensity of second peak as a function of brake duration, see Additional file 1 for the protocol of the experiment. Panels H and I magnitude of the second and the first peak of IKK and NF-*κ*B activity (nuclear NF-*κ*B and IKKa) calculated from average over 500 cells. In numerical experiment presented in Panel I, the receptor activity coefficient *k*_*a *_was elevated 10 fold before second TNF*α *pulse.

Although several studies [23,25,45,50,51] have clarified the relationship between time profile of IKK activity and NF-*κ*B oscillations, less is known about the regulation of IKK activity itself. In response to TNF stimulation, IKK is rapidly activated and then inactivated. As demonstrated by the experimental findings [24] and modeling [23], an important mechanism for this inactivation is mediated by the NF-*κ*B – A20 negative feedback loop. Here, a short pulse of TNF*α *results in a brief peak of IKKa followed by peak in nuclear NF-*κ*B and burst of A20 mRNA and protein. The newly synthesized A20 attenuates IKK activity, making the cell temporarily less sensitive to subsequent TNF*α *stimulation. As described earlier, A20 acts via direct and indirect mechanisms. Directly it binds to the regulatory subunit of IKK, speeding transformation of IKKa into the inactive, hyperphosphorylated IKK (represented as IKKi). Indirectly, it ubiquitinates RIP, a necessary component of the active receptor complex leading to its specific proteasomal degradation, thereby lowering the average receptor activity. An interesting question arises: How much time is needed for the system to recover, i.e., how long should be the break after a brief TNF*α *pulse to allow for the equal response to subsequent pulse? In order to answer the question we performed an experiment (see Additional file 1 for details of experimental protocol) in which the population of 3T3 cells is stimulated by two brief TNF*α *pulses (5 min., at 20 ng/ml) spaced at various times apart ranging from 30 to 180 min. This saturating TNF*α *concentration makes the individual cell responses well synchronized, allowing for reliable population analysis, Fig 3. The IKK kinase activity is measured 5 min. after the second pulse, and the IKK activity of the second peak relative to the first is expressed as a function of break duration.

The experiment helped us to determine the parameters governing the NF-*κ*B – A20 – IKK negative feedback loop, Figs 3G and 3H. Although after a short break the system did not respond, full recovery of the system was observed after 2.5 hours. Surprisingly, when the duration of the break was extended to 3 hours the second peak of IKK activity was higher than the first one. One could interpret this result as the evidence that after 2.5 to 3 hours the cytoplasmic A20 concentration decreases to pretreatment values and some other protein is elevating IKK activity. One of the candidates could be TRAF2, which is NF-*κ*B responsive and is a constituent of the TNFR1 receptor complex [52]. To test this hypothesis we assume that at second peak the activity of TNF*α *bound receptors is elevated 10 fold, Fig. 3I, and in fact this modification resulted in an elevated IKK profile similar to the experimental findings. However, since we do not find this a strong enough verification, we have not introduced this modification to the current model.

### Cell respond to stimulations by a wide range of TNF*α *doses

**Figure 4:**
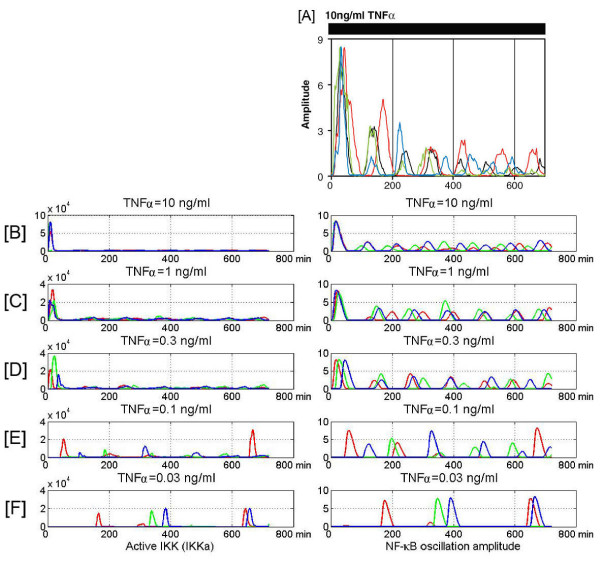
Single cell experiment and simulation. Panel A: Nuclear-to-cytoplasmic NF-*κ*B oscillations measured by Nelson et al. [33] shown for four SKNAS cells (marked by different colors) stimulated by 10 ng/ml TNF*α*. Panels B to F: IKKa and NF-*κ*B oscillation amplitude resulting from model simulations, respectively for TNF*α *dose: 10, 1, 0.3, 0.1 and 0.03 ng/ml. In each panel we show three single cell simulations, marked by different colors.

We performed the single cell stochastic numerical simulations (SCSNS) of our model to analyze the individual cell responses to persistent stimulation in a broad range of TNF*α *doses. We assumed that there were 1000 TNFR1 receptors on cell surface, and each of these receptors might be independently activated or inactivated, and that the activation rate was proportional to the TNF*α *concentration, which was kept constant during the experiment. At a high TNF*α *dose (above 1 ng/ml) the receptor activation rate is high and most of cells are activated in the first few minutes after the TNF*α *stimulation begins. As a result, the first peak of IKK activity and the first peak of NF-*κ*B nuclear to cytoplasmic oscillation are well synchronized among cells. Synchronization of subsequent peaks of NF-*κ*B oscillations decreases due to the stochastic processes of activation of TNFR1 receptors and A20 and I*κ*B*α *genes. Such behavior well agrees with Nelson et al. [33] single cell experiment on SKNAS cells, Fig 4A versus 4B.

**Figure 5:**
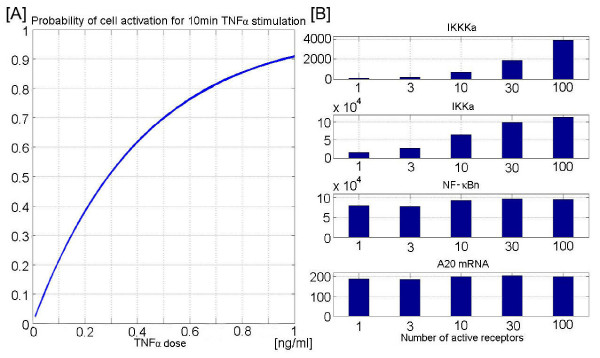
Characteristics of cell activation as a function of dose. Panel A: Probability of cell activation (at least one receptor activation) for 10 min. stimulation as a function of TNF*α *dose. Panel B: Cell activity measured as an average height of the IKKKa, IKKa, nuclear NF-*κ*B, and A20mRNA peak resulting from 10 min. activity of 1, 3, 10, 30, or 100 receptors.

For low doses (0.1 and 0.03 ng/ml, Figs 4E and 4F), the activation of each cell is typically due to the activation of single receptor or a small number of receptors and thus the first response time varies between cells. As a result, the NF-*κ*B oscillations are not synchronized at all. Moreover, for the lowest dose (0.03 ng/ml, Fig 4F) the time span between subsequent oscillations varies significantly. The last is due to the fact that when TNF*α *molecules dissociate the cell become unstimulated. It is however possible that dissociated TNF*α *molecule is immediately captured by other TNFR1 receptors (or transiently by TNFR2 receptors) of the same cell. This effect would keep the once stimulated cell in semi-periodic oscillatory mode for the longer time. Interestingly, the amplitude of the second and subsequent oscillations is higher for the low TNF*α *dose than for the high dose. This is due to the fact that at a low TNF*α *dose the IKK activation proceeds not as fast: the first peak of IKKa is smaller than for a high dose (Fig. 5) and more IKKn remains to be activated. As a result the system at low dose stimulation exhibits subsequent IKKa pulses followed by high NF-*κ*B oscillations (Fig. 4).

### Activation of a single TNFR1 receptor complex (trimer) turns on the NF-*κ*B regulatory module

**Figure 6:**
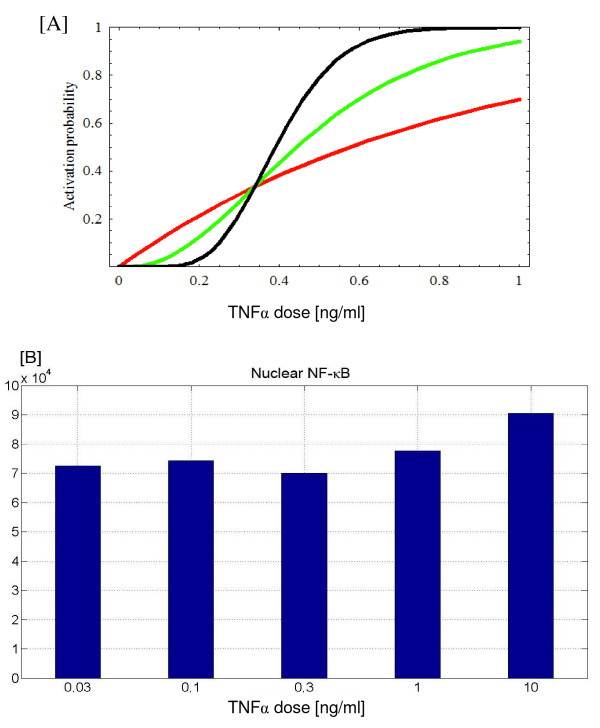
Model predictions for pulse low dose TNF*α *stimulation. Panel A: Probability of cell activation during 5 min. long TNF*α *stimulation as a function of dose, model prediction (red) versus threshold hypothesis: green (3 receptors), black (10 receptors). Panel B: Average height of nuclear NF-*κ*B peak calculated for subpopulation of N = 500 of activated cells (at least with one receptor activated) after 5 min. long TNF*α *stimulation as a function of TNF*α *dose.

Mentioned earlier, the ability of the cell to respond to femtomolar TNF*α *concentrations suggests that NF-*κ*B regulatory module may be turned on by the single TNF*α *trimer activating one TNFR1 receptor (receptor trimer). According to our model the lower TNF*α *dose results in lower probability of cell activation, but not in a much weaker response in the responding cells (Figs. 5A and 6B). We have chosen the receptors activation coefficient such that 90% of cells are activated (have at least one receptor active) in the first 10 minutes of TNF*α *stimulation by the dose of 1 ng/ml, which may be treated as a saturation dose, until the persistent stimulation is considered. This is in agreement with experimental observations in which responses to TNF*α *doses of 1 ng/ml or 10 ng/ml are almost identical. Below this dose, the individual cell responses become asynchronous and at any given moment the population is a mixture of responsive and nonresponsive cells.

Because the NF-*κ*B regulatory network has the amplification-saturation pathway build in, the final cell response measured at the level of NF-*κ*B responsive genes is about the same in the case when the single receptor (trimer) is activated as when 100 receptor complexes are activated. To see how cell's response depends on the number of active receptors, one can perform the following numerical experiment. Let us assume that at *t *= 0, a given number N (1, 3, 10, 30 or 100) of receptors is activated and that these receptors remain active for 10 min. We traced the average heights of the peaks of active IKKK, active IKK, nuclear NF-*κ*B, and A20 mRNA, Fig. 5B. We thus observed four levels of signal saturation; first three are connected with the transduction-amplification pathway, the last one with gene regulation. According to our model the fivefold decrease in peak IKK activity when we pass from 100 to 1 active receptor, does not result in much lower NF-*κ*B oscillation amplitude. This is in agreement with bulk of experimental data suggesting that peak IKK activity at high and intermediate TNF*α *dose of 10 or 1 ng/ml is much higher than needed for efficient I*κ*B*α *degradation. Specifically, Lee et al. [24] demonstrated (for TNF*α *dose of 10 ng/ml) that a very low IKK activity tail is sufficient to maintain oscillations in I*κ*B*α *level, and that a higher IKK activity tail, typical for A20 deficient MEFs, totally suppress I*κ*B*α *accumulation. This result was confirmed by quantitative data of Werner et al. [50] who stimulated A20 deficient, immortalized 3T3 cells by a 45 minute long TNF*α *pulse at a dose of 1 ng/ml. They found that the peak of IKK activity (same for wild type and A20 -/- cells) was followed by tail of fivefold lower magnitude but that this was enough to keep most of NF-*κ*B in the nucleus. The fact that NF-*κ*B remains nuclear implies high I*κ*B*α *synthesis rate and thus high degradation rate, so it may not accumulate to uptake NF-*κ*B back to cytoplasm.

The high sensitivity of NF-*κ*B responses to low dose TNF*α *stimulation has been already reported by Cheong et al. [45] who, based on experiments with decreasing TNF*α *dose from 10 to 0.01 ng/ml, concluded that NF-*κ*B amplitude is a logarithmic function of TNF*α *dose: It decreases only twice as the doses changes from 10 to 0.1 ng/ml. The effect we reported in Figs. 5B and 6B is even more dramatic, the NF-*κ*B amplitude remains almost independent of the TNF*α *dose assuming that only the activated cells are considered. The single cell model we constructed suggests that the decrease of NF-*κ*B amplitude shown by Cheong et al. [45] (Fig. 1) is at least partially due to pure synchronization of cells. As recently experimentally demonstrated by Sillitoe et al. [53] the averaging causes that NF-*κ*B pulses appear much smaller than they are. Sillitoe et al. [53] experiment was performed for TNF*α *dose of 10 ng/ml, but even at that dose (at which one may expect cells are relatively well synchronized) the first peak, amplitude of the average trajectory is about twice smaller than single cell amplitudes, and this difference is larger for the second peak at which cells are less synchronized. In addition, at the smallest dose of 0.01 ng/ml studied by Cheong et al. [45], which corresponds to only 1/4 of TNF*α *trimer per cell volume, we observe the effect of averaging over responsive and nonresponsive cells, which lowers the amplitude of average NF-*κ*B trajectory.

There is still not enough experimental data at single cell level to uniquely determine how each step of transduction amplification cascade contributes to amplification. Cheong et al. [45] attempted to address this question based on population data and found that a 100 fold decrease of TNF*α *dose from 10 to 0.1 ng/ml results in a fourfold decrease of IKK activity peak and in a twofold decrease of NF-*κ*B amplitude. As already discussed, the population data can be quite misleading, and in fact to fit to this data Cheong et al. [45] had to assume time dependent IKK activation and inactivation rates. Such approach seems artificial and cannot be applied to more general protocols of TNF*α *stimulation (e.g., to pulse-pulse stimulation).

As discussed above, the constructed model has the property that the stable activity of a single receptor is sufficient to turn on the NF-*κ*B regulatory module and trigger the transcription of NF-*κ*B dependent genes. Although the experimental data suggests that the a very limited number of active receptors is needed to induce NF-*κ*B activity, there is no evidence that activity of a single receptor is sufficient. One may expect that there exists a threshold of the number of active receptors (receptor complexes) needed for cell activation. In the case of T-cells stimulation, it was experimentally demonstrated [15] that just three major peptide histocompatibility complexes are required to induce T-cell cytotoxic activity. T-cell receptor signalling involves MAPK kinase cascade [54], which can convert graded inputs into switch-like outputs [55]. Both MAPK and MAPKK require two phosphorylations to become fully activated, and this dual phosphorylation is a source of nonlinearity in signal processing, which together with saturation (with a strong signal all MAPK is phosphorylated) results in a switch-like output.

Since we did not identify any simple mechanisms defining the threshold here, we followed the simplest possibility and assumed that amplification provided by the TNFR1-IKKK-IKK-I*κ*B*α *cascade is high enough to cause that activation of a single receptor is sufficient to induce cell activity. To rule out or confirm the competitive threshold hypothesis it is worth to consider a series of single cell experiments, in which cells would be stimulated for 5 min. with a decreasing TNF*α *dose, and measure the fraction of responding cells. Pulse stimulation would help localize activation and response to relatively short time periods.

According to our model, the probability that a single receptor is activated by a TNF*α *stimulation lasting for time *t *at a concentration *C *is equal to

(1) *pr*_1_(*C*) = 1 - exp(-*k*_*b*_*Ct*),

where *k*_*b *_is the receptor activation rate. Thus, if activation of a single receptor is sufficient to activate the cell, then the fraction of responding cells *F *would decrease approximately linearly with TNF*α *concentration *C *for low concentrations *C*. If however, at least *n *receptors must be activated in order to activate the cell then the probability of cell activation would be equal to *pr*_*n*_

(2) $$pr_{n}(C)=1-\sum_{i=0}^{n-1}\left(\begin{array}{c} N\\ i \end{array}\right)\times {\left[1-\exp\left(-k_{bn}Ct\right)\right]}^{i}\exp{\left(-k_{bn}Ct\right)}^{N-i},$$

where *N *is total number of receptors and *k*_*bn *_is the new hypothetical TNF*α *binding constant. In such case the fraction of responding cells F would decrease faster, approximately as *C*^*n *^(for small *C*). In Fig. 6A we compare model predictions (*n *= 1, red line) against threshold hypothesis (*n *= 3, green line) and (*n *= 10, black line) for *N *= 1000. Activation probability functions *pr*_3_(*C*) and *pr*_10_(*C*) are normalized by adjusting activation rates *k*_*b*3 _≃ 5.03 *k_b_* and *k*_*b*10 _≃ 20.79 *k*_*b*_, what assures that all *pr*_*n*_(*C*) functions intersect at the same point at which *pr*_*n*_(*C*) = 1/3.

The single pulse, low dose, experiment would also enable us to determine the "minimum cell response" as the average (over subpopulation of responding cells) NF-*κ*B oscillation amplitude for very small *C*. According to our model (Fig. 6B) such a minimum response exists and moreover it is high enough to assure unequivocal expression of NF-*κ*B responsive genes in majority of responding cells.

## Conclusion

Stochastic gene activation (leading to the burst of proteins) and stochastic cell activation (leading to the massive NF-*κ*B nuclear translocation) leads to what might be called "stochastic robustness" in cell regulation. If a given gene is activated, a large burst of proteins is produced, in order to assure a sufficient level of activity of these proteins. Stochastic robustness assures the minimal response to the signal. Decreasing magnitude of the signal mostly reduces the probability of response, which leads to a smaller fraction of responding cells. This may be a useful strategy: If the TNF*α *signal is low, some cells respond by a massive NF-*κ*B translocation, whereas some do not respond at all. It helps to avoid ambiguity, such as when a small nuclear concentration of NF-*κ*B leads to activation of an undefined fraction of NF-*κ*B responsive genes. Such an ambiguous response might do more harm than good. In fact the all-or-none responses arising due to ultrasensitivity and saturation or bistability (typically connected with positive feedbacks) have been reported for various signalling elements: TCR signaling [54], Xenopus p42 MAPK cascade [55], transduction cascade JNK in oocytes [56], and Lac operon, e.g. [57]. The growing evidence of bistability in various system may suggest that it is a good strategy for regulation at the tissue level.

Stochastic robustness allows cells to respond differently to the same stimulation, but makes their individual responses better defined. Both effects could be crucial in early immune response: Diversity in cell responses causes the tissue defense to be harder to overcome by relatively simple programs coded in viruses and other pathogens. The more focused single cell responses help cells to decide their individual fates such as proliferation or apoptosis.

**Figure 7:**
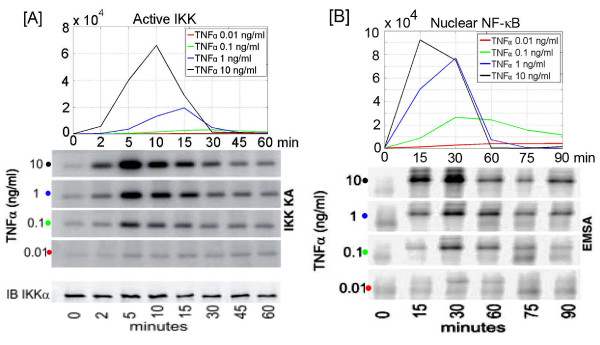
Average IKK and NF-*κ*B activity (nuclear NF-*κ*B and IKKa) as function of TNF*α *dose, model versus Cheong et al. 2006 experiment [45]. Panels A: IKKa and IKK kinase activity, Panel B: nuclear NF-*κ*B calculated as average of N single cell simulation: N = 50 for TNF*α *dose equal 10 ng/ml, N = 100 (1 ng/ml), N = 500 (0.1 ng/ml) and N = 2500 (0.01 ng/ml). and EMSA assays of nuclear NF-*κ*B. To compare our predictions to experimental data, we rescale the time coordinate, i.e. we calculate the values of corresponding functions in the same time points as in the experiment. Despite the qualitative agreement between the model and the experiment shown there is substantial difference in IKK activity peak timing and smaller difference in NF-*κ*B profile timing for 10 ng/ml dose. In fact there is also large discrepancy between different experiments; here the IKK activity peak is observed for 5 min., while in [50] for 10 min. and in [62] for 15 min.

The stochastic model proposed by us explains the mechanisms of TNF*α *cell activation at nanomolar concentrations when the number of molecules of activation factor per cell is very limited. In the example of TNF*α *diffusion in the tissue, considered by Cheong et al. [45], concentration of 0.01 ng/ml or 200 fM corresponds to less than one TNF*α *trimer per cell. In such case population consists of responding and nonresponsive cells, and the output observed at the population level reflects averaging these two subpopulations. At low dose stimulation no individual cell evolves like the average and the average does not correspond to any biological process. This means that the model build to follow the average trajectory in general may not be correct. Despite this fact, the experimental average may be useful when compared with the average of many single cell stochastic simulations. Predictions from our model agree qualitatively with large set of population data (see Additional file 3: Figs S1, S2, S3 and S4) including the Cheong et al. [45] experiment in which IKK and NF-*κ*B activity were measured across a wide range of TNF*α *concentrations, Fig. 7.

The proposed model supports hypothesis that at nanomolar concentrations NF-*κ*B activity results from binding of single TNF*α *trimers. Such hypersensitivity of NF-*κ*B regulatory module may be the only way to detect and respond to single viruses invading cells and to allow for cytokine extracellular signaling.

## Methods

### Stochastic NF-*κ*B model with two feedback loops

The model applied involves two-compartment kinetics of NF-*κ*B and its inhibitors A20 and I*κ*B*α*, and allows analyzing single cell responses to TNF*α *stimulation of arbitrary and time dependent intensity, in both wild type and A20 deficient cells. It combines our previous model [35] with the signal transduction-amplification cascade, which transmits the signal from the TNFR1 receptors, [30], to the NF-*κ*B-I*κ*B*α*-A20 regulatory module. The single event of receptor activation is amplified by the three-step signal transduction cascade involving activation of kinase named IKKK (IKK activating kinase) and IKK [30]. Biologically, there are at least two kinases involved in this process: MEKK3 [58,59] and TAK1 [59-61]. Shim et al. [61] showed that TAK1 mediates IKK activation in TNF*α *and IL-1 signaling pathways. Homozygous mutants (Tak1^*m*/*m*^) do not show NF-*κ*B activity under TNF*α *stimulation, and exhibit lowered NF-*κ*B activity than the wild type cells in response to IL-1. Nho et al. [58] found that silencing MEKK3 by siRNA reduces IKK activity. These two findings and the experiment by Blonska et al. [59] suggest that TAK1 is recruited to the TNFR1 complex via RIP and likely cooperates with MEKK3 to activate NF-*κ*B in TNF*α *signaling. In our model we mimic this possibly complex transduction mechanism by a single entity: IKKK. To accomplish the above, we assume that IKKK migrates toward the receptor and is activated at a receptor (transformation from IKKKn to IKKKa). Active IKKKa molecules activate IKK molecules (transformation from IKKn to IKKa) which in turn phosphorylate I*κ*B*α *molecules leading to their ubiquitination and degradation. It is assumed that the total number of IKKK and IKK molecules (as well as of the NF-*κ*B molecules) is constant; i.e., their degradation is balanced by production, but both terms are omitted in the mathematical representation. The IKKK molecules may exist in one of two states,

• native, neutral, IKKKn, specific to unstimulated resting cells without, and

• active IKKKa.

The activity of IKKK is very transient, i.e., it is activated rapidly (with the maximum rate 1/s from single active receptor) and inactivated with the half time of about 1 min.

IKK complexes, consisting of catalytic subunits IKK*α *and IKK*β *and regulatory subunits IKK*γ*, may exist in one of four states,

• native neutral (denoted by IKKn), specific to unstimulated resting cells.

• active (denoted by IKKa), arising from IKKn via phosphorylation of serines 177 and 181 of IKK*β *subunits [62] upon the IKKKa induced activation,

• inactive (denoted by IKKi), but different from the native neutral form, arising from IKKa possibly due to overphosphorylation, and

• transient between IKKi and IKKn, also inactive, denoted by IKKii.

The activation pathway considered, which involves kinases IKKK and IKK, resembles the one introduced by Park et al. [30]. The main difference is that we take into account the inactivation of IKKa is due to its transformation to the state IKKi different from the native state IKKn. As a result, in contrast to Park et al. model [30], the tonic TNF*α *stimulation induces transient IKK activity in agreement with the experimental data, Figs 6 and S2.

In resting cells, the unphosphorylated I*κ*B*α *binds to NF-*κ*B and sequesters it in an inactive form in the cytoplasm. IKKa mediated phosphorylation of I*κ*B*α *leads to it degradation and releases the main activator NF-*κ*B, which then enters the nucleus and triggers transcription of the two inhibitors and numerous other genes. The newly synthesized I*κ*B*α *leads NF-*κ*B out of the nucleus and sequesters it in the cytoplasm.

IKK inactivation is controlled by the second inhibitor A20, which like I*κ*B*α*; is strongly NF-*κ*B responsive [22]. The exact mechanism of A20's action is still not fully resolved. Here we assume that A20 acts in two ways: (1) It initiates degradation of RIP, the key component of the TNFR1 receptor complex [63], which attenuates the activity of receptors, and (2) it directly associates with IKKγ [64], enhancing IKKa conversion (this conversion takes place also in A20 deficient cells but at a slower rate) to catalytically inactive form IKKi. The exact mechanism of IKK inactivation remains unresolved: According to Delhase et al. [62] IKK inactivates via autophosphorylation of serines in IKK*β *C-terminal region. However, recently Schomer-Miller et al. [65] found that this autophosphorylation does not diminish IKK activity and suggested that phosphorylation of serines 740 and 750 in NBD/γBD domain of IKK*β *may have a regulatory role and that their phosphorylation may downregulate IKK activity. The form IKKi spontaneously converts into IKKn through inactive intermediate forms collectively denoted by IKKii. The number of these forms may be large since there are at least 16 serine residues in IKK*β *[65], which may be involved in regulation of IKK activity. This intermediate step is introduced in the model to account for the delay needed to process the inactivated form IKKi into native state IKKn. This effect is manifested in A20-/- cells (at persistent or long lasting TNF*α *stimulation) as a downregulation of IKK activity at about 30 min. followed by a higher plateau (Figs. S2 and S3, reproduced after [24] and [50] experiments). According to our model in the first minutes of high dose TNF*α *stimulation most of IKKn is used up so the IKK activation rate is low, only after some IKKn is recovered via intermediated form IKKii, the activation rate and the level of IKKa may increase.

The inhibitor I*κ*B*α *migrates between the nucleus and cytoplasm and forms complexes with NF-*κ*B molecules. The nuclear I*κ*B*α*|NF-*κ*B complexes quickly migrate into the cytoplasm. The second inhibitory protein A20 is considered only in the cytoplasm where it triggers the inactivation of IKK. It is assumed that the transformation rate from IKKa into IKKi is the sum of the constant term and a term proportional to the amount of A20.

The transcriptional regulation of A20 and I*κ*B*α *genes is governed by the same rapid elongation regulatory mechanism with a rapid coupling between NF-*κ*B binding and transcription. The mechanisms for NF-*κ*B dependent regulation of I*κ*B*α *and A20 are based on the control of transcriptional elongation. In this situation, stalled RNA polymerase II is rapidly activated by NF-*κ*B binding to enter a functional elongation mode, and requires continued NF-*κ*B binding for reinitiation. This is represented in our model by tight coupling of NF-*κ*B binding to mRNA transcription. We assume that all cells are diploid, and both A20 and I*κ*B*α *genes have two potentially active homologous copies, each of which is independently activated due to binding of NF-*κ*B molecule to a specific regulatory site in gene promoter. Following [1,3,8,35] and others we made the simplifying assumption that each gene copy may exist only in one of two states; active and inactive. When the copy is active the transcription is initiated at a high rate, when the copy is inactive transcription is inhibited. The gene copy becomes inactive when the NF-*κ*B molecule is removed from its regulatory site due to the action of I*κ*B*α *molecules, which bind to DNA-associated NF-*κ*B, exporting it out of the nucleus.

In this work, as in our recent papers [35] we follow the method proposed by Haseltine and Rawlings [66] and split the reaction channels into fast and slow. We consider all reactions involving mRNA and protein molecules as fast and the reactions of receptors and genes activation and inactivation as slow. Fast reactions are approximated by the deterministic reaction-rate equations, whereas slow reactions are considered stochastic.

According to the above, the mathematical model consists of 15 ordinary differential equations (ODEs) accounting for

• formation of the (I*κ*B*α*|NF-*κ*B) complexes,

• IKKK and IKK kinase activation and inactivation

• IKKa driven I*κ*B*α *phosphorylation,

• A20, I*κ*B*α *and phospho-I*κ*B*α *proteins degradation

• transport between nucleus and cytoplasm, and

• transcription and translation.

All the substrates are quantified by the numbers of molecules. The upper-case letters denote substrates or their complexes. Nuclear amount is represented by subscript *n*, while subscript *c *denoting amount of substrate in the cytoplasm is omitted, to simplify the notation. Amounts of the mRNA transcript of A20 and I*κ*B*α *are denoted by subscript *t*:

### Notation guide

• IKKn – neutral form of IKK kinase,

• IKKa – active form of IKK,

• IKKi – inactive form of IKK,

• IKKii – inactive intermediate form of IKK,

• *K*_*NN *_– total number of IKK = IKKn+IKKa+IKKi+IKKii molecules (assumed to be constant in time)

• IKKKa – amount of active form of IKKK,

• IKKKn – amount of neutral form of IKKK,

• *K*_*N *_– total number of IKKK=IKKKn+IKKKa molecules (assumed to be constant in time)

• I*κ*B – cytoplasmic amount of I*κ*B*α*,

• I*κ*B_*n *_– nuclear I*κ*B*α*,

• I*κ*B_*t *_– I*κ*B*α *mRNA transcript,

• I*κ*B_*p *_– phosphorylated cytoplasmic I*κ*B

• NF*κ*B|I*κ*B – cytoplasmic (NF-*κ*B|I*κ*B*α*) complexes

• NF*κ*B|I*κ*B_*p *_– phosphorylated cytoplasmic I*κ*B*α *complexed to NF*κ*B

NF*κ*B|I*κ*B_*n *_– nuclear (NF*κ*B|I*κ*B*α*) complexes

• *TNF *– TNF*α *concentration,

• *G*_*IκB *_– discrete random variable, state of I*κ*B*α *gene,

• *G*_*A*20 _– discrete random variable, state of A20 gene,

• *k*_*v *_= *V/U *– ratio of cytoplasmic to nuclear volume.

• *B *– number of active receptors, *M *– total number of receptors (assumed to be constant in time)

#### IKKK in active state (IKKKa)

The first term describes IKKK kinase activation, i.e. transformation from IKKKn (amount of which is IKKK*n *= *K*_*N *_- *IKKKa*) due to action of active receptors *B*(*t*), whose activity is attenuated by A20. The second term describes spontaneous inactivation of the kinase

(3) $$\frac{d}{dt}IKKKa(t)=k_{a}\times k_{a20}/(k_{a20}+A20)\times B(t)\times (K_{N}-IKKKa(t))-k_{i}\times IKKKa(t).$$

#### IKK in the natural state IKKn

The first term describes IKKn recovery from the intermediate form IKKii (amount of which is *IKKii *= *K*_*NN *_- *IKKn *- *IKKa *- *IKKi*), whereas the second term describes depletion of IKKn due to its transformation into IKKa mediated by IKKKa

(4) $$\frac{d}{dt}IKKn(t)=k_{4}\times (K_{NN}-IKKn-IKKa-IKKi)-k_{1}\times IKKKa(t)\times IKKn(t).$$

#### IKK in the active state IKKa

The first term represents transition from IKKi to IKKa mediated by IKKKa, whereas the second term represents depletion of IKKa due to its transformation into inactive form IKKi mediated by A20

(5) $$\frac{d}{dt}IKKa(t)=k_{1}\times IKKKa(t)\times IKKn(t)-k_{3}\times IKKa(t)\times (k_{2}+A20(t))/k_{2}$$

#### IKK in the inactive state IKKi

The first term corresponds to the formation of inactive IKKi from IKKa by A20 mediated inactivation, whereas the second term describes transformation into IKKii

(6) $$\frac{d}{dt}IKKi(t)=k_{3}\times IKKa(t)\times (k_{2}+A20(t))/k_{2}-k_{4}\times IKKi(t).$$

#### Phospho-I*κ*B*α *(I*κ*B_*p*_)

The first term describes I*κ*B*α *phosphorylation due to catalytic action of IKKa, the second term catalytic degradation of phosphorylated I*κ*B*α*

(7) $$\frac{d}{dt}I\kappa B_{p}(t)=a_{2}\times IKKa(t)\times I\kappa B(t)-t_{p}\times I\kappa B_{p}(t).$$

#### Phospho-I*κ*B*α *complexed to NF-*κ*B (NF*κ*B|I*κ*B_*p*_)

The first term describes I*κ*B*α *phosphorylation (in complexes with NF-*κ*B) due to the catalytic action of IKKa, the second term catalytic degradation of phosphorylated I*κ*B*α *(NF-*κ*B is recovered)

(8) $$\frac{d}{dt}(NF\kappa B|I\kappa B_{p})(t)=a_{3}\times IKKa(t)\times (NF\kappa B|I\kappa B)(t)-t_{p}\times (NF\kappa B|I\kappa B_{p})(t).$$

#### Free cytoplasmic NF-*κ*B

The first two terms represents liberation of free NF-*κ*B due to degradation of I*κ*B*α *in (I*κ*B*α*|NF-*κ*B) complexes and its depletion due to formation of these complexes. The third term accounts for liberation of NF-*κ*B due to degradation of phospho-I*κ*B*α*. The last term describes transport of free cytoplasmic NF-*κ*B to the nucleus,

(9) $$\begin{array}{lll} \frac{d}{dt}NF\kappa B(t) & = & c_{6a}\times (NF\kappa B|I\kappa B)(t)-a_{1}\times NF\kappa B(t)\times I\kappa B\alpha (t)\\ & & +t_{p}\times (NF\kappa B|I\kappa B\alpha_{p})(t)-i_{1}\times NF\kappa B(t). \end{array}$$

#### Free nuclear NF-*κ*B

The first term describes transport into the nucleus. The second term represents depletion of free nuclear NF-*κ*B due to the association with nuclear I*κ*B*α *and is adjusted, by multiplying the synthesis coefficient *a*_1 _by *k*_*v *_= *V/U*, to the smaller nuclear volume resulting in a larger concentration,

(10) $$\frac{d}{dt}NF\kappa B_{n}(t)=i_{1}\times NF\kappa B(t)-a_{1}\times k_{v}\times I\kappa B_{n}(t)\times NF\kappa B_{n}(t).$$

#### A20 protein

Described by its mRNA synthesis and constitutive degradation,

(11) $$\frac{d}{dt}A20(t)=c_{4}\times A20_{t}(t)-c_{5}\times A20(t).$$

#### A20 transcript

The first term stands for NF-*κ*B inducible synthesis, while the second term describes degradation of the A20 transcript,

(12) $$\frac{d}{dt}A20_{t}(t)=c_{1}\times G_{A20}-c_{3}\times A20_{t}(t).$$

#### Free cytoplasmic I*κ*B*α *protein

The first term accounts for IKKa induced phosphorylation, the second for NF-*κ*B binding. The second line describes I*κ*B*α *synthesis and the constitutive degradation of I*κ*B*α*. The last two terms represent transport into and out of the nucleus,

(13) $$\begin{array}{lll} \frac{d}{dt}I\kappa B(t) & = & -a_{2}\times IKKa(t)\times I\kappa B(t)-a_{1}\times I\kappa B(t)\times NF\kappa B(t)\\ & & +c_{4a}\times I\kappa B_{t}(t)-c_{5a}\times I\kappa B(t)\\ & & -i_{1a}\times I\kappa B(t)+e_{1a}\times I\kappa B_{n}(t). \end{array}$$

#### Free nuclear I*κ*B*α *protein

The first term corresponds to I*κ*B*α *association with nuclear NF-*κ*B (adjusted, by multiplying the synthesis coefficient *a*_1 _by *k*_*v*_, for the smaller nuclear volume resulting in larger concentration), and the last two terms represent the transport into and out of the nucleus,

(14) $$\frac{d}{dt}I\kappa B_{n}(t)=-a_{1}\times k_{v}\times I\kappa B_{n}(t)\times NF\kappa B_{n}(t)+i_{1a}\times I\kappa B(t)-e_{1a}\times I\kappa B_{n}(t).$$

#### I*κ*B*α *transcript

The first term stands for NF-*κ*B inducible synthesis, whereas the second term describes degradation of I*κ*B*α *transcript

(15) $$\frac{d}{dt}I\kappa B_{t}(t)=c_{1}\times G_{I\kappa B}-c_{3}\times I\kappa B_{t}(t).$$

Note that Eq. (15) (as well as 12) naturally produces saturation in transcription speed. When the nuclear amount of regulatory factor NF-*κ*B is very large, then the binding probability is much larger than the dissociation probability, and the gene state would be *G*_*a *_= 2 for most of the time. In such case the transcription would proceed at a maximum rate, 2*c*_1_.

#### Cytoplasmic (I*κ*B*α*|NF-*κ*B) complexes

The first line describes formation of the complexes due to I*κ*B*α *and NF-*κ*B association and their degradation. The first term in the second line represents phosphorylation of the (I*κ*B*α*|NF-*κ*B) complexes due to the catalytic activity of IKKa. The last term represents transport of the complex from the nucleus,

(16) $$\begin{array}{lll} \frac{d}{dt}(I\kappa B|NF\kappa B)(t) & = & a_{1}\times NF\kappa B(t)\times I\kappa B(t)-c_{6a}\times (NF\kappa B|I\kappa B)(t)\\ & & -a_{3}\times IKKa(t)\times (NF\kappa B|I\kappa B)(t)+e_{2a}\times (NF\kappa B|I\kappa B_{n})(t). \end{array}$$

#### Nuclear (I*κ*B*α*|NF-*κ*B) complexes

Described by their formation due to I*κ*B*α *and NF-*κ*B association (adjusted, by multiplying the synthesis coefficient *a*_1 _by *k*_*v*_, to the smaller nuclear volume resulting in larger concentration) and their transport out of the nucleus,

(17) $$\frac{d}{dt}(NF\kappa B|I\kappa B_{n})(t)=a_{1}\times k_{v}\times I\kappa B_{n}(t)\times NF\kappa B_{n}(t)-e_{2a}\times (NF\kappa B|I\kappa B_{n})(t).$$

### Propensities of receptors and genes activation and inactivation

The receptors activate and inactivate independently with activation propensity $r_{r}^{b}(t)$ proportional to the TNF*α*(*t*) concentration (which may be time dependent) and inactivation propensity $r_{r}^{d}$ constant

(18) $$\begin{array}{cc} r_{r}^{b}(t)=k_{b}\times TNF(t), & r_{r}^{d}=k_{d}. \end{array}$$

We assume that both A20 and I*κ*B*α *genes have two homologous copies independently activated due to NF-*κ*B binding, and inactivated due I*κ*B*α *mediated removal of NF-*κ*B molecules, and that binding and dissociation propensities *r*^*b*^(*t*) and *r*^*d*^(*t*), respectively, are equal for each copy:

(19) *r*^*b*^(*t*) = *q*_1 _× *NFκB*_*n*_(*t*),     *r*^*d*^(*t*) = *q*_2 _× *IκBα*_*n*_(*t*).

The state of gene copy *G*^*i *^(*i *= 1, 2) is *G*^*i *^= 1 whenever NF-*κ*B is bound to the promoter regulatory site, and *G*^*i *^= 0 when the site is unoccupied. As a result the gene state *G *= *G*^1 ^+ *G*^2 ^can be equal to 0, 1 or 2. In this approximation the stochasticity of single cell kinetics solely results from discrete regulation of receptors activity and transcription of A20 and I*κ*B*α *genes.

In model computations, the amounts of all the substrates are expressed as the numbers of molecules. Since we use the ODE's to describe most of the model kinetics, amounts of molecules are not integer numbers, but since these numbers are in most cases much greater than 1, such description is reasonable.

### Numerical implementation

The numerical scheme implemented follows that of [35]:

(1) At simulation time *t*; for given states $G_{A20}=G_{A20}^{1}+G_{A20}^{2}\text{ and }G_{I\kappa B}=G_{I\kappa B}^{1}+G_{I\kappa B}^{2}$ of the A20 and I*κ*B*α *genes, and number of active (bound) receptors *B *(*M *is the total number of receptors) we calculate the total propensity function *r*(*t*) of occurrence of any of the activation and inactivation reactions

(20) $$r(t)=r_{r}^{b}(M-B)+r_{r}^{d}B+r_{A20}^{b}(t)(2-G_{A20})+r_{I\kappa B}^{b}(t)(2-G_{I\kappa B})+r_{A20}^{d}(t)G_{A20}+r_{I\kappa B}^{d}(t)G_{I\kappa B}.$$

(2) We select two random numbers *p*_1 _and *p*_2 _from the uniform distribution on [0, 1].

(3) Using the fourth order MATLAB solver we evaluate the system of 15 ODEs accounting for fast reactions, until time *t *+ *τ *such that

(21) $$\log(p_{1})+\int_{t}^{t+\tau }r(s)ds=0.$$

(4) There are 6 potentially possible different reactions:

• receptor may be activated or inactivated. Typically in time course there are many inactive receptors which may activated and active receptors which may be inactivated, but since the receptors are assumed to be identical it is not important which one of them changes its state.

• NF-*κ*B may bind to or dissociate from any of two alleles of A20 and I*κ*B*α *genes.

In this step we determine which one of 8 potentially possible reactions occurs at time *t*+*τ *using the inequality

(22) $$\sum_{i=1}^{k-1}r_{i}(t+\tau )<p_{2}r(t+\tau )\leq \sum_{i=1}^{k}r_{i}(t+\tau )$$

where *r*_*i*_(*t *+ *τ*), *i *= 1,...,6 are individual reaction propensities and *k *is the index of the reaction to occur.

(5) Finally time *t *+ *τ *is replaced by *t*, and we go back to item (1).

In all simulations before TNF*α *stimulation starts, we simulate a resting cell for time *t *randomly chosen from the interval of 15 to 25 hours in order to get equilibrated and randomized initial conditions. As shown in [35] the resting cells oscillate. Due to natural degradation of I*κ*B*α*, some NF-*κ*B molecules may occasionally enter the nucleus and activate the A20 or I*κ*B*α *gene, which results in bursts of A20 and I*κ*B*α *mRNAs and proteins. As a result the initial (at *t *= 0) level of A20 or I*κ*B*α *mRNA and protein differs across the population, which influences future cells evolution.

In Figs. 8 and 9 we present evolution of selected variables during the persistent TNF*α *stimulation at high (10 ng/ml) and low (0.1 ng/ml) dose. We show both a single cell evolution and the average over 100 cells. The average is used to validate the model based on population data (Figs 3, 4, 7 and Figs S1, S2, S3, S4 in Additional file 3).

**Figure 8:**
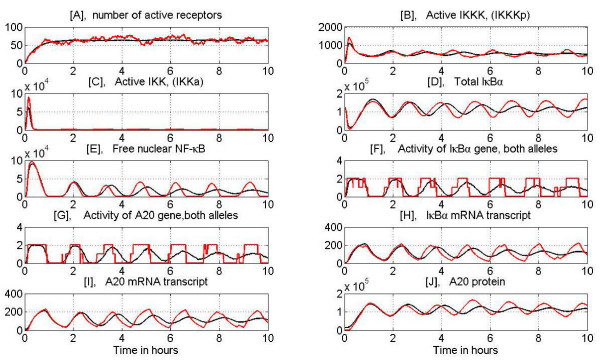
Evolution of main variables during persistent, 10 hours long stimulation with high dose TNF*α *= 10 ng/ml. Red line – typical single cell simulation, black line – average over 100 cells. Panel A: Number of active receptors, Panel B: IKKKa level, Panel C: IKKa level, Panel D: Total I*κ*B*α*, Panel E: Nuclear NF-*κ*B, Panel F: I*κ*B*α *gene activity, Panel G: A20 gene activity, Panel H: I*κ*B*α *mRNA, Panel I: A20 mRNA, Panel J: A20 protein.

**Figure 9:**
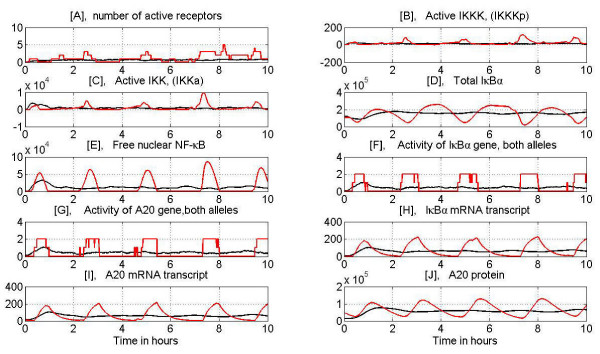
Evolution of main variables during persistent, 10 hours long stimulation with low dose TNF*α *= 0.1 ng/ml, see Fig. 8 for description. Note, that at low dose stimulation first IKKa peak is smaller than for high dose but the subsequent peaks are well pronounced. As a result subsequent NF-*κ*B oscillations are higher for low than high dose.

To compare model predictions with Nelson et al. experiment [33] we calculate the NF-*κ*B nuclear to cytoplasmic oscillation amplitudes assuming that 40% of total pool of NF-*κ*B does not oscillate, Fig. 4. We also convert ratio of nuclear to cytoplasmic amounts to ratio of light intensities, making use of the fact that nucleus is smaller than cytoplasm. These two operations do not influence the character of the oscillations but only their amplitude. The non-oscillatory fraction (not taken in account in the model) remains in the cytoplasm even after all I*κ*B*α *is degraded. This fraction is observed also in population experiments (data not shown) and it possibly consists of NF-*κ*B bound to other I*κ*B*α *isoforms and of NF-*κ*B molecules with nuclear localization sequence damaged.

### Model fitting and parameters

The model is intended. cells showing oscillations in NF-*κ*B nuclear-to-cytoplasmic localization under persistent TNF*α *stimulation. We do not to fit the parameters to any given single experiment but we intuitively choose the set of parameters which produces qualitative agreement with a major subset of existing data. Quantitative agreement is not possible because there is a substantial discrepancy mostly in IKK activity and NF-*κ*B peak timing between experiments). Because of a large number of undetermined parameters, this is a tedious task, but in our opinion it is better to produce a model in qualitative agreement with the current and previous experiments, than a model perfectly fitted to a single experiment with limited set of data. We based our choice of parameters on both single cell and population experiments. In the letter case, to compare our model with experiments, we average a large number of single-cell stochastic simulations. This procedure is much more time consuming that comparing the deterministic model with the population data, but as already shown (in the case of low dose TNF*α *stimulation) the population data do not correspond to any biological process, and thus constructing the model fitted to such a data is not justified.

We applied the following method of choosing the values of parameters:

1) Start from a reasonable set of parameters, which produces a correct steady state in the absence of TNF*α *signal.

2) Proceed with the signal initiated by TNF downstream the autoregulatory loops.

3) Iterate item 2 until the fit to all the data is satisfactory.

The TNF*α *signal first causes activation of receptors then transformation of IKKKn into IKKKa which catalyses transformation of IKKn into IKKa. In turn, IKKa catalyses degradation of cytoplasmic (I*κ*B*α*|NF-*κ*B); enabling the free NF-*κ*B to enter the nucleus. Once NF-*κ*B builds up in the nucleus it upregulates the transcription of the A20 and I*κ*B*α *genes. After being translated, A20 facilitates transformation of IKKa into IKKi and blocks the receptors, while I*κ*B*α *enters the nucleus, binds to NF-*κ*B and leads it into cytoplasm. As stated in item 2, we first fit the coefficients regulating IKK activation (using data on IKK activity), then the coefficients regulating degradation of the cytoplasmic (I*κ*B*α*|NF-*κ*B) and I*κ*B*α *degradation and so forth. If there were no feedback loops in the pathway, the proposed method would be quite efficient, but, since they exist, it is necessary to iterate the signal tracing several times, until the fit is satisfactory. Once a satisfactory set of parameters is found, we observe that this set of parameters is not unique. This ambiguity is mainly caused by the lack of measurements of absolute values of protein or mRNA amounts. The action exerted by some components of the pathway onto the rest of the pathway is determined by their amounts multiplied by undetermined coupling coefficients. Hence, once we have a good set of parameters, we may obtain another one using a smaller coupling coefficient and by proportionately enlarging the absolute level of the component. Since not all parameters may be determined based on existing data we have assumed values of part of parameters mostly based on our intuition and fitted the remaining ones. By such approach we show how much information can be inferred from available experimental data. Ambiguity in parameter determination leads to significant differences between parameters of our model and the corresponding parameters chosen by others groups of researcher. Values of all model parameters are listed in discussed in detail in Additional file 2.

The validation of the proposed model, is based on our data (see Fig. 3) in addition to [33] (see Fig. 4), [45] (see Fig. 7), [28] (see Additional file 3: Fig. S1), [24] (see Additional file 3: Fig. S2), [50] (see Additional file 3: Fig S3), [34] (see Additional file 3: Fig S4) and [62] experiments. Typical experimental data at our disposal consist of measurements made at time points that are not uniformly distributed. Therefore, to compare our predictions to experimental data, we rescale the time coordinate, i.e. we calculate the values of corresponding functions in the same time points as in the experiment, and then to guide the eye we connect the resulting discrete points by straight-line segments, thus obtaining a saw-like graph.

## Abbreviations

A20 (TNFAIP3), TNF alpha inducible protein 3; I*κ*B*α*, inhibitor of *κ*B *α *subunit; IKK, I*κ*B kinase; IKKK, IKK kinase; IRAK1 interleukin-1 receptor-associated kinase 1; MEKK3, mitogen-activated protein kinase kinase kinase 3; NF-*κ*B, nuclear factor-*κ*B; RIP, receptor interacting protein; TAK1, transforming growth factor-*β*-activated kinase 1; TNF*α*, tumor necrosis factor *α*; TNFR, TNF receptor; TRAF2, tumor necrosis factor receptor associated factor 2.

## Competing interests

The author(s) declares that there are no competing interests.

## Authors' contributions

TL, ARB and MK discussed the working hypothesis and wrote the paper. TL constructed and fitted the mathematical model. PP and MK discussed the mathematical model. PP wrote MATLAB programs. KP performed final numerical simulations and prepared the figures. ARB performed the IKK kinase activity experiment. All authors read and approved the final manuscript.

## Supplementary Material

Additional file 3 Supplementary figures S1, S2, S3 and S4. Validation of the proposed model based on experiments by Hoffmann et al. [28], Fig. S1; Lee et al. 2000 [24], Fig. S2; Werner et al. [50], Fig S3; and Nelson et al. [34], Fig S4. Click here for file

Additional file 1 Protocol of IKK kinase activity measurements. Click here for file

Additional file 2 Model parameters and their justification. Values and discussion of model parameters. Click here for file
