# Supplementary material for: Single TNFα trimers mediating NF-κB activation: stochastic robustness of NF-κB signaling
Source: BMC Bioinformatics. 2007 Oct 9;8:376. doi: 10.1186/1471-2105-8-376 (PMC2222661; doi:10.1186/1471-2105-8-376)
Supplement: Additional file 3 — Supplementary figures S1, S2, S3 and S4. Validation of the proposed model based on experiments by Hoffmann et al. [28], Fig. S1; Lee et al. 2000 [24], Fig. S2; Werner et al. [50], Fig S3; and Nelson et al. [34], Fig S4. [file 1471-2105-8-376-S3.pdf]

Additional file 3: Supplementary figures S1, S2, S3 and S4

T. Lipniacki et al

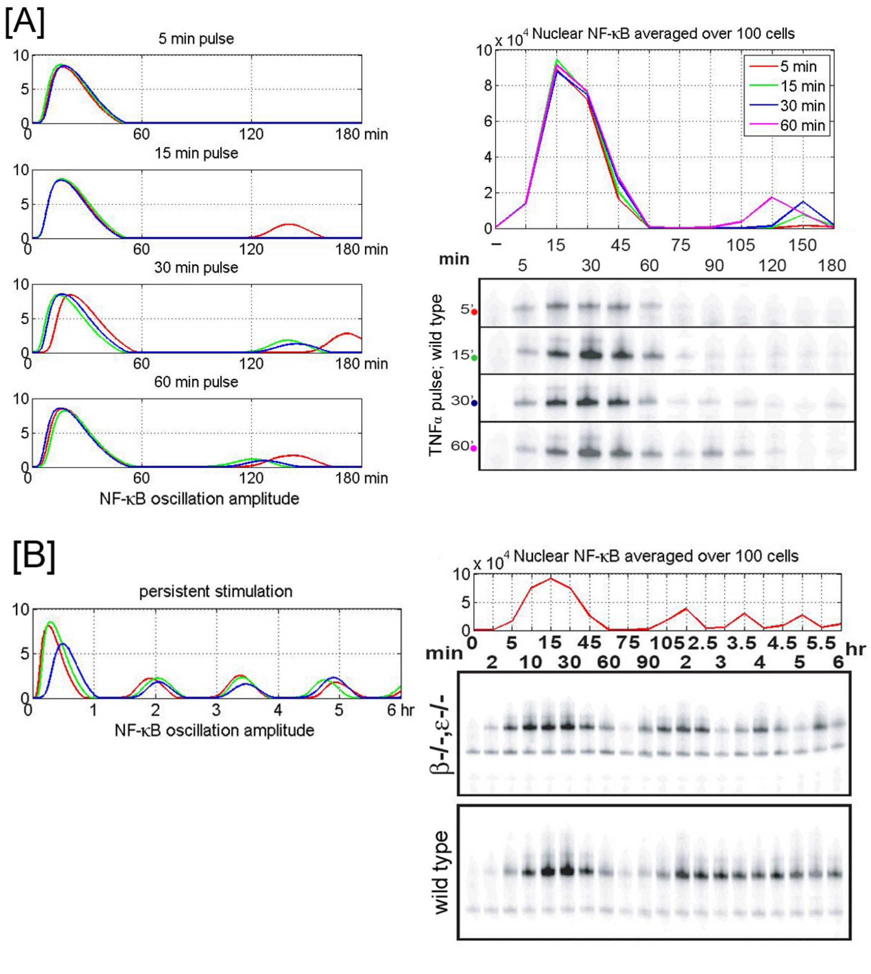

Fig. S1. Pulse and persistent stimulation by a high TNFα dose (10ng/ml). Amplitude of nuclear to cytoplasmic NF-κB oscillations in single cells, and nuclear NF-κB averaged over 100 cells compared with the experiment on mouse embryonic fibroblast (Hoffmann et al. *Science* 2002, **298**:1241-1245). Panel A, 5, 15, 30 and 60 min. long pulse stimulation. Panel B, persistent stimulation. Nuclear NF-κB averaged over 100 cells compared with data from IκBε and IκBβ deficient and wild-type fibroblast (Hoffmann et al. *Science* 2002, **298**:1241-1245).

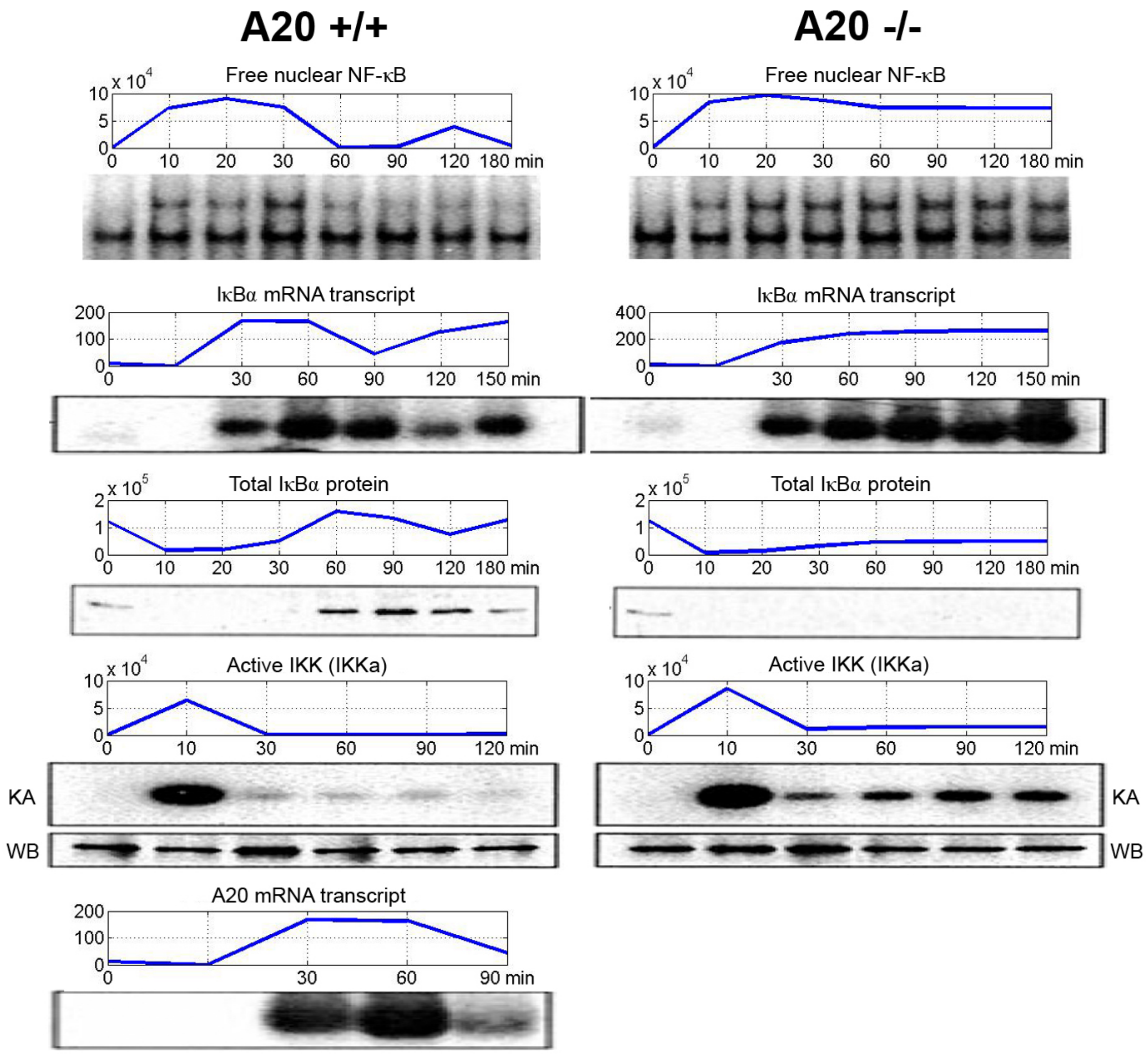

Fig. S2. The role of A20 negative feedback control in persistent TNFα stimulation. Model predictions versus experiment for wild type and A20-/- mouse embryonic fibroblast (Lee et al. *Science* 2000, **289**:2350-2354).

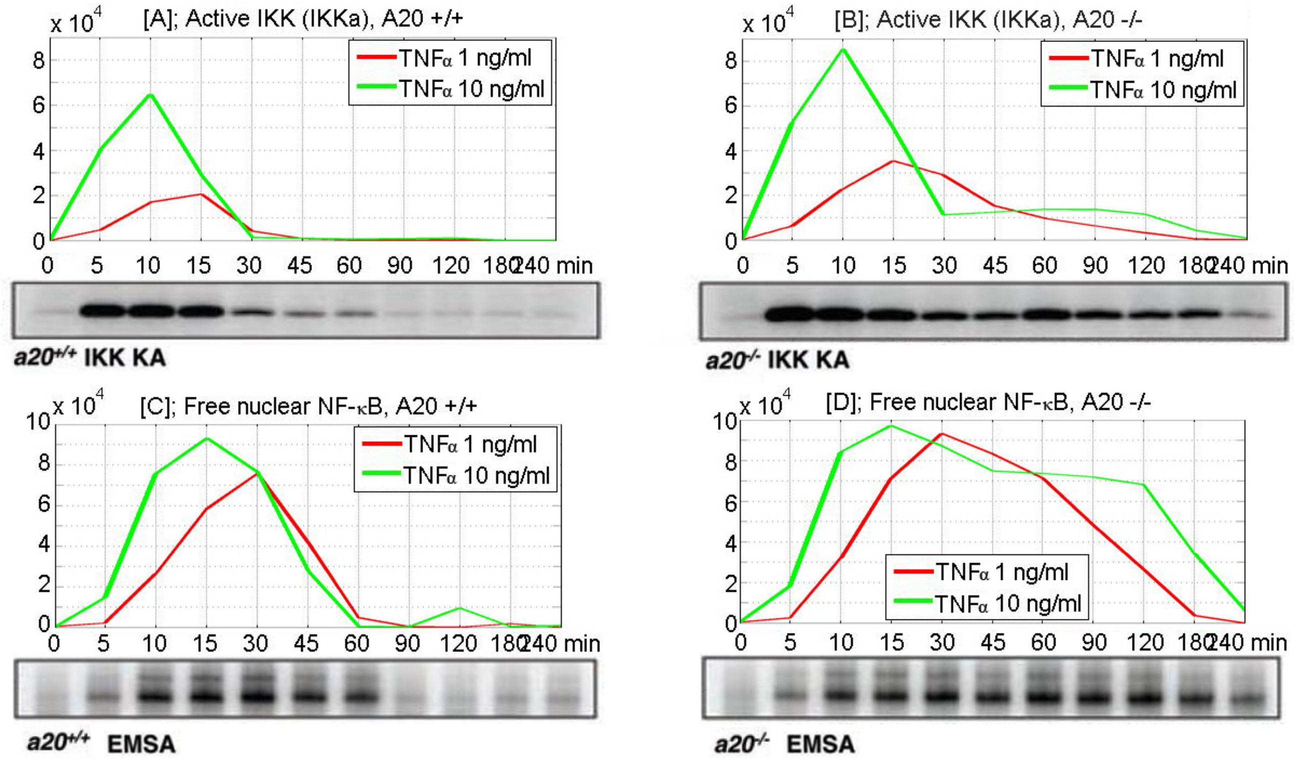

Fig. S3. The role of A20 negative feedback control in pulse TNFα stimulation. Experiment on 3T3 (Werner et al. *Science* 2005, **309**:1857-1861) stimulated by a 45 min. pulse of 1= ng/ml TNFα, versus model predictions for TNFα=1ng/ml and TNFα=10ng/ml (a better agreement with the experiment is achieved when in the numerical simulation TNFα concentration is set at 10ng/ml). Panel A, IKK activity of wild type cells in response to 45-min stimulation. Panel B, IKK activity of A20-/- cells. Panel C, nuclear NF-κB of wild-type cells. Panel D, nuclear NF-κB of A20-/- cells.

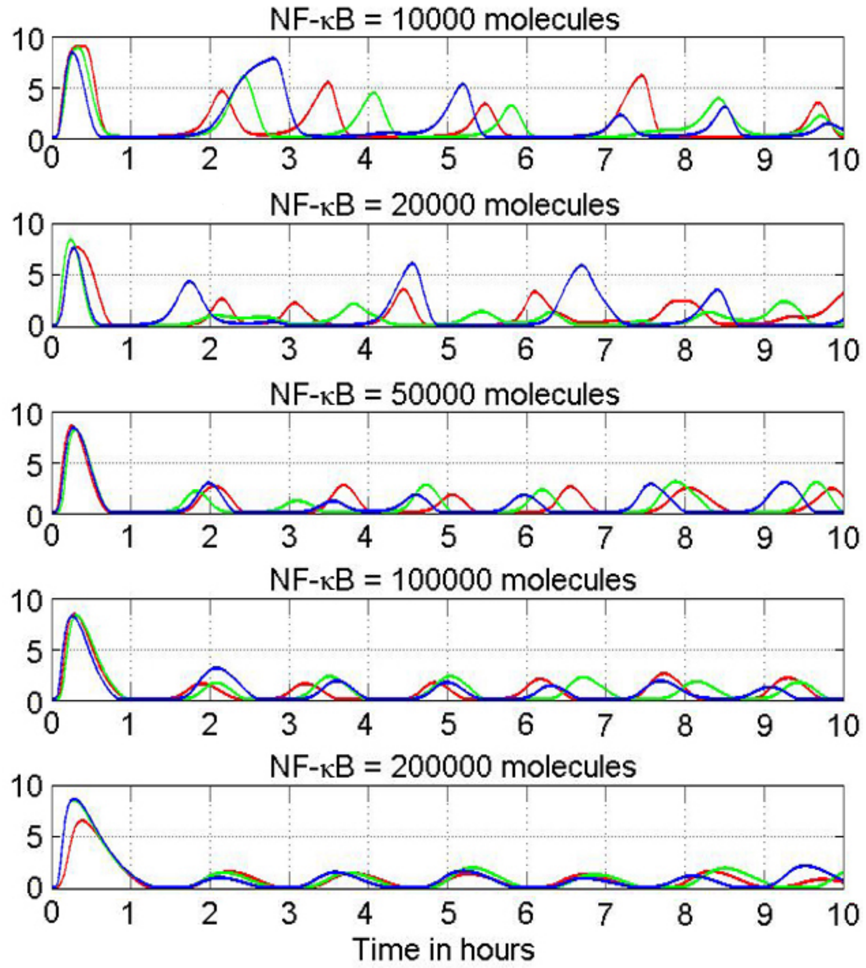

Fig. S4. Nuclear to cytoplasmic NF-κB oscillations during persistent treatment by 10ng/ml TNF for 5 levels of total NF-κB, 10000, 20000, 50000, 100000, 200000 molecules. The figure shows weak dependence of oscillation period to the total amount of NF-κB, in agreement with the single cell experiment (Nelson et al. *Science* 2005, **308**:52b).
