# Supplementary material for: Single TNFα trimers mediating NF-κB activation: stochastic robustness of NF-κB signaling
Source: BMC Bioinformatics. 2007 Oct 9;8:376. doi: 10.1186/1471-2105-8-376 (PMC2222661; doi:10.1186/1471-2105-8-376)
Supplement: Additional file 1 — Protocol of IKK kinase activity measurements. [file 1471-2105-8-376-S1.pdf]

Additional file 1: Protocol of IKK kinase activity measurements

T. Lipniacki et al.

3T3 cells were stimulated for 5 min. with TNFα (20 ng/ml) in culture medium (DMEM, 10% FBS). The cells were then washed with pre-warmed PBS twice, and fresh culture medium was replaced. At the indicated times, cells were stimulated with a second pulse of TNFα (20 ng/ml, 5min). Cells were washed, medium was replaced. 5 min. later, cells were washed in ice cold PBS, scraped in PBS with protease inhibitors and phosphatase inhibitors (1 mM Na_3VO_4, 10 mM NaF, 10 mM $Na_4P_2O_7$, and 1 mM PMSF), and precipitated by centrifugation (2000 rpm) in a microfuge for 3 min. at 4 ^{o}C. Cells were then resuspended in 1 mL of Lysis Buffer (50 mM HEPES, pH 7.6, 150 mM NaCl, 1 mM EDTA, 1.5 mM $MgCl_2$, 10 % Glycerol, 1% IGEPAL). After incubation on ice for 20 min., 500 μg of protein were immunoprecipitated with 2 μg rabbit polyclonal anti-IKKa/b Ab (Santa Cruz Biotech, SC-7607) overnight at 4 ^{o}C with rocking. IKK was then precipitated with the addition of 50 μL of Protein A sepharose slurry for 2-3 h at 4 ^{o}C. Beads were washed twice with Lysis Buffer, and twice with Kinase Buffer (20 mM HEPES, pH 7.6, 10 mM $MgCl_2$, 50 mM NaCl, 20 mM β-glycerophosphate, 100 μM Na_3VO_4, 20 μM ATP, 10 μg/ml aprotinin, 2 mM DTT). Each precipitate was adjusted to 20 μL in Kinase Buffer, and 4μCi of γ-^{32}P-ATP and 2 μg of GST-IκBα(1-51). Kinase reactions were incubated for 30 min. at 30 ^{o}C with gentle shaking. The products were then denatured in SDS-PAGE loading buffer, fractionated by 10% SDS-PAGE, transferred to PVDF membranes and exposed to autoradiography. Quantitation of signal was performed by exposure to Molecular Dynamics Phosphorimager cassette.
