# Supplementary material for: Single TNFα trimers mediating NF-κB activation: stochastic robustness of NF-κB signaling
Source: BMC Bioinformatics. 2007 Oct 9;8:376. doi: 10.1186/1471-2105-8-376 (PMC2222661; doi:10.1186/1471-2105-8-376)
Supplement: Additional file 2 — Model parameters and their justification. Values and discussion of model parameters. [file 1471-2105-8-376-S2.pdf]

Additional file 2: Model parameters and their justification

T. Lipniacki et al.

Table 1. Values of assumed and fitted parameters

Symbol	Values	Units	Description	Comments
W_0	2×10^{-12}	l	cell volume	Assumed [1]
$k_v = V/U$	5		cytoplasmic to nuclear volume	Assumed
k_b	4×10^{-6}	$s^{-1}ml/ng$	receptor activation rate	Assumed
k_f	6×10^{-4}	s^{-1}	receptor inactivation rate	Fitted [3]
q_1	1.5×10^{-7}	s^{-1}	NF-κB driven activation of IκBα and A20 genes	Fitted
q_2	10^{-6}	s^{-1}	IκBα mediated NF-κB dissociation from IκBα and A20 sites	Assumed
KN	10^4		total number of IKKK molecules	Assumed
KNN	$2 \cdot 10^5$		total number of IKK molecules	Assumed
M	10^3		total number of receptors	Assumed
NF-κB$_{tot}$	10^5		total number of NF-κB molecules	Assumed [1]
k_a	10^{-4}	s^{-1}	IKKK activation rate	Assumed
k_i	0.01	s^{-1}	IKKK inactivation rate	Assumed
c_1	0.1	s^{-1}	inducible A20 and IκBα mRNA synthesis	Assumed
c_3	0.00075	s^{-1}	A20 and IκBα mRNA degradation	Fitted [2]
c_4	0.5	s^{-1}	A20 and IκBα translation rate	Fitted
c_5	0.0005	s^{-1}	A20 degradation rate	Fitted
k_{a20}	10^4		A20 TNFR1 block	Fitted
k_2	10^4		IKKa inactivation due to A20	Fitted
k_1	5×10^{-6}	s^{-1}	IKKn activation rate	Fitted
k_3	0.003	s^{-1}	IKKa inactivation rate	Fitted
k_4	0.0005	s^{-1}	IKKii transformation	Fitted
a_1	5×10^{-7}	s^{-1}	IκBα–NFκB association	Assumed [4]
a_2	10^{-7}	s^{-1}	IKKa mediated IκBα phosphorylation	Fitted
a_3	5×10^{-7}	s^{-1}	IKKa mediated (NFκB$\vert$IκBα) phosphorylation	Fitted
tp	0.01	s^{-1}	degradation of P-IκBα and P-IκBα bounded to NF-κB	Fitted
c_{5a}	0.0001	s^{-1}	degradation of IκBα	Assumed [5]
c_{6a}	0.00002	s^{-1}	spontaneous degradation of IκBα bounded to NF-κB	Assumed [5]
i_1	0.01	s^{-1}	NF-κB nuclear import	Fitted
e_{2a}	0.05	s^{-1}	IκB$\alpha\vert$NF-κB nuclear export	Fitted
i_{1a}	0.002	s^{-1}	IκBα nuclear import	Fitted
e_{1a}	0.005	s^{-1}	IκBα nuclear export	Fitted

Parameters justification and discussion

Cell dimensions

W_0 - cell volume: we adopted value the $2 \times 10^{-12} l$ after Carlotti et al., [1]. The value W_0 does not appear explicitly in the model, but is needed to translate concentrations into numbers of molecules per cell. The same value was used in our previous studies [9], [10], and in [12].

$k_v = V/U$ - cytoplasmic to nuclear volume: typically cytoplasm is 3-10 times the volume of the nucleus [14], [15], depending on cell type. We assumed the value $k_v = 5$, as in our previous works [9], [10], while Cheong et al. [12] assumed $k_v = 7$. In original Hoffmann et al. model [13], implicitly $k_v = 1$ was assumed and which was followed by [16], [17], [18], [19]. In further considerations (if not otherwise stated) to translate concentration into number of molecules we use cytoplasmic volume of $(5/6) \times 2 \times 10^{-12} l = 1.67 \times 10^{-12} l$ and nuclear volume of $1/6 \times 2 \times 10^{-12} l = 0.33 \times 10^{-12} l$. Thus the cytoplasmic concentration of 1nM corresponds to 1000 molecules, while same nuclear concentration corresponds to 200 molecules.

Coefficients of the TNFR1-IKKK-IKK-IκBα transduction pathway

This transduction pathway transmits TNFα signal downstream causing IκBα phosphorylation and degradation and subsequent nuclear NF-κB translocation. The coefficients of this pathway have to be fitted simultaneously, since in most cases the change of one of coefficients may be compensated by the change of others. Since we do not have direct measurements on TNFR1 receptors and IKKK activity, the first constraint is IKK activity (measured most accurately by Delhase [7]) and then ubiquitous data on IκBα degradation and NF-κB nuclear translocation. The action of the pathway is attenuated by A20, which is NF-κB responsive, which makes fitting of pathway coefficients difficult.

Important developments in modeling of TNFR1-IKKK-IKK-IκBα signaling are due to Park et al. [8]. The strongest discrepancy between Park et al. [8] and our models is in the IKK activity profile. According to Park et al. model [8] TNFα stimulation results in sharp rise of active IKK which reaches the plateau, while according to our model IKK activity is transient with high peak at about 10-15 min. of TNFα stimulation followed by a very low tail. In the case of A20-deficient cells the tail is higher, but still much lower than the peak. The transient character of IKK activity was observed first by Delhase et al. [7] in HeLa cells and then by Lee et al. [20] in mouse fibroblasts, and then by Werner et al. [16]. This transient nature of IKK activity is possibly due not to the phosphatase dephosphorylation but rather, as shown by Delhase et al. [7] to overphosphorylation.

$M = 1000$ – total number of TNFR1 receptors, assumed. Variation of this parameter may be compensated by k_b. The number of TNFR1 receptors per cell may vary significantly between cell lines [21], e.g. there are about 3000 TNFR1 per cell for HeLa [3], and about 10000 for Histiocytic lymphoma (U-937) cells [21], but much less for B-cell lymphoma (Raji) cells.

$k_b = 4 \times 10^{-6} \mathrm{s}^{-1}\mathrm{ml/ng}$ – receptor activation rate. We have chosen this activation coefficient to assure that 90% of cells are activated (have at least one receptor active) in first 10 min. of TNFα stimulation with the dose of 1ng/ml, which may be considered as a saturation dose. The receptor activation rate should not be confused with TNFα binding rate. Binding of TNFα trimer initiates receptor TNFR1 trimerization and formation of an active receptor complex in a multistep process

involving binding of RIP and TRAF2. Park et al. [8] considered several reversible processes leading to the receptor activation.

$k_f = 6 \times 10^{-4}\text{s}^{-1}$– receptor inactivation rate, it corresponds to $t_{1/2} = 20\,\text{min}$. This is in accordance with Grell [3], who found that TNFα trimers dissociate from TNFR1 receptors with a half time of 33 min., while the internalization time is of the order of 10 to 20 min. Park et al. [8] assumed $k_f = 2 \times 10^{-3}\text{s}^{-1}$.

$KN = 10^4$ – total number of IKKK molecules, assumed. There is substantial freedom in choosing this parameter, i.e. a different choice of KN may be compensated by other parameters of the transduction pathway. Park et al. [8] assumed IKKK concentration of 0.045μM (what gives 45,000 molecules).

$k_a = 10^{-4}\text{s}^{-1}$ (IKKK activation rate) and $k_i = 0.01\text{s}^{-1}$(IKKK inactivation rate) are assumed. The value of k_a together with that of KN implies that single receptor activates at most one IKKK molecule per second. High k_i causes the IKKK-IKK transduction pathway to have small inertia. Park et al. [8] assumed TRAF IKKK association rate corresponding to k_a equal to 10 $\mu\text{M}^{-1}\text{s}^{-1}$ (which corresponds to 10^{-5}s^{-1}). Our estimation of parameters k_a, k_i and k_1 (see below) is based on values of the corresponding parameters of the well studied MAPK pathway [25].

$KNN = 2 \times 10^5$ – total number of IKK molecules, assumed. In our previous study [10] the same total number of IKK molecules was maintained by the balance of production and degradation terms. In the original Hoffmann et al. model [13], only the active IKK was considered and its initial concentration was assumed to be 0.1 μM (what gives 100,000 molecules). In Cheong et al. [12] time and TNFα dose dependent IKK activation/inactivation rates are used. At highest TNFα dose considered, concentration of active the IKK reaches 0.2 μM. In Kearns et al. [17], the initial concentration of active IKK of 0.8μM (800,000 molecules) is assumed at the start of the stimulation phase. Park et al. [8] assumed total IKK concentration of 0.06μM.

$k_1 = 5 \cdot 10^{-6}\text{s}^{-1}$– IKKn activation rate. This value was fitted, it implies that one IKKKa molecule activates at most one IKKn molecule per second. Since IKKn was activated directly by TNFα in previous our models [9], [10], the meaning of k_1 is different than previously. Park et al. [8] assumed IKKK IKK association rate corresponding to k_1 equal to 10 $\mu\text{M}^{-1}\text{s}^{-1}$ (which corresponds to 10^{-5}s^{-1}).

$k_3 = 0.003\text{s}^{-1}$ –IKKa inactivation rate, fitted. In [9] and [10] $k_3 = 0.0015\text{s}^{-1}$.

$k_4 = 0.0005\text{s}^{-1}$–IKKii transformation, fitted. This coefficient represents two rates: transformation from IKKi to IKKii and from IKKii to IKKn. It was fitted based on [20] and [16], showing elevation of the IKK activity at about 1h in A20-/- cells, which in our model is due to recovery of IKKn through intermediate form IKKii.

$k_{a20} = 10^4$ – A20 mediated TNFR1 block; this value was fitted based on pulse-pulse and A20 knockout experiments. It implies that when the number of A20 molecules equals 10^4 the activity of TNFα bound receptors is twice lower than in the absence of A20.

$k_2 = 10^4$ – IKKa inactivation due to A20; this value was fitted based on pulse-pulse and A20 knockout experiments. It implies that when the number of A20 molecules equals 10^4, inactivation of IKKa proceeds twice faster than in the absence of A20. The new value k_2 corresponds to former $k_3/k_2 = 3 \times 10^4$ [10]. Thus the influence of A20 onto IKKa inactivation is higher, but A20 is less abundant because it degrades faster (at the rate c_5) and its mRNA also degrades faster (at the rate c_3) than in [10].

$a_2 = 10^{-7}\text{s}^{-1}$ (IKKa mediated IκBα phosphorylation) and $a_3 = 5 \times 10^{-7}\text{s}^{-1}$ (IKKa mediated phosphorylation of IκBα|NF-κB complexes), fitted. IκBα phosphorylation proceeds through its

binding to IKKa, but since these complexes are very unstable, we assumed that IKKa directly phosphorylates free IκBα and complexed with NF-κB with rates corresponding to formation of these unstable complexes. Thus $a_2 = 10^{-7}\text{s}^{-1}$ corresponds to IKKa–IκBα synthesis rate of 0.1 $\mu M^{-1}\text{s}^{-1}$, while $a_3 = 5\times10^{-7}\text{s}^{-1}$ corresponds IKKa–IκBα|NF-κB synthesis rate of 0.5 $\mu M^{-1}\text{s}^{-1}$. In [13], [18], [16] these rates are respectively 0.0225 $\mu M^{-1}\text{s}^{-1}$ and 0.185 $\mu M^{-1}\text{s}^{-1}$, but the kinetics of active IKK was very different, as said the initial concentration was assumed to be 0.1 μM, and then IKK was freely degrading with the half time of 2.3 h in the presence of TNFα, or with the half time of 5 min. in the absence of TNFα.

At high TNFα dose IKKa pulse reaches 70,000 molecules which yields IκBα phosphorylation rate of 0.007 s^{-1}, and IκBα|NF-κB phosphorylation rate of 0.035 s^{-1}, which allows for almost total IκBα degradation in first 10-15 min. of TNFα stimulation.

The capability of activation of NF-κB by a single TNFα molecule follows from high amplification of a signal by TNFR1-IKKK-IKK-IκBα transduction cascade. Specifically the magnitude of this amplification is determined by coefficients k_f, KN, k_a, k_i, KNN, k_1, k_3, a_2, a_3. As already said there is substantial freedom in choice of these parameters since the change of one parameter may be compensated by the change of others. For example the expected number of IKKK molecules activated by a single receptor is $k_a \cdot KN/k_f$ and expected number of IKK molecules activated by IKKKa molecule is $k_1 \cdot KNN/k_i$; the actual number of active IKKKa and IKKa resulting from activity of single receptor is however lower due to their rapid inactivation governed respectively by coefficients k_i and k_3.

Gene activation/inactivation and transcription/translation rates

$q_1 = 1.5\cdot10^{-7}\text{s}^{-1}$(NF-$\kappa$B driven activation of I$\kappa$B$\alpha$ and A20 genes), and $q_2 = 10^{-6}\text{s}^{-1}$ (IκBα mediated NF-κB dissociation from IκBα and A20 sites); these values are adopted after our previous work [10]. They imply fast gene activation (with $t_{1/2}$ of order of one minute) when most of the 100,000 NF-κB molecules are in the nucleus, and almost immediate turning off of NF-κB dependent genes when the bulk of freshly synthesized IκBα moves into nucleus.

Since estimation of transcription and translation coefficients is controversial, we discuss it here in detail. The total amount of synthesized protein is proportional to the product of mRNA transcription and translation rates, thus one has some freedom in determining c_1 and c_4. We assumed a likely value of $c_1 = 0.1$mRNA/s (transcription speed per gene copy) and then we fitted value of $c_4 = 0.5$ protein/(mRNA×s) trying to keep both values with accordance to current knowledge.

In previous work [10] we assumed $c_1 = 0.075\ \text{s}^{-1}$, while in [9] where the transcription rate was proportional to NF-κB concentration in cytoplasm we assumed the value of 0.16 s^{-1} as the upper limit reached when all NF-κB is in the nucleus. This limit was based on following estimation: The typical transcription speed in animal cells is of the order of 40 nucleotides (nt) per second (Levin, [11] p. 129). A single gene, however, can be read by a number of RNA polymerases simultaneously (see e.g. Levin [11]). Assuming that spacing between subsequent RNA polymerases is of the order of 250 nt one obtains the transcription initiation frequency of 40 (nt/s)/250nt = 0.16s^{-1}. Cheong et al. [12] assumed the upper limit of transcription as 0.55s^{-1} based on transcription speed of 55nt/s and spacing between mRNA polymerases of 100nt [27], [28]. In our opinion Cheong et al. [12] estimation gives the highest reasonable limit. The mRNA synthesis rate has been measured by Femino et al. [26] for β-actin by single RNA transcript visualization as 4 mRNA/min.

Much higher transcription speeds of the order of tens of mRNA per second follow from Hoffmann et al. model [13] and subsequent works [16], [17], [18], [19]. Specifically in [13] transcription

speed is a second order function of nuclear NF-κB concentration (which reaches $0.07\mu M$ in their model simulations), thus the fitted transcription parameter tr2 $= 1.65 \times 10^{-2}\mu M^{-1}s^{-1}$ implies transcription speed tr2$\times$(NF-κB$_n$)$^2/2 = 8 \times 10^{-5}\mu M$ s^{-1}, or 40mRNA/s per gene copy. In [17], [18], [19] about the same values of tr2 are assumed. In [16] transcription speed is a third order function of nuclear NF-κB concentration, and thus fitted their coefficient rs_an= $0.132 \times \mu M^{-2}$s^{-1} together with the highest nuclear concentration of $0.07\mu M$ considered, implies transcription speed of 23 mRNA/s per gene copy.

The translation coefficient $c_4 = 0.5$s^{-1} is adopted after our first [9] and second study [10] where it was justified by the estimation of translation speed of 15 amino acid/s and spacing between ribosomes of 30 amino acids (or 90 nt). This estimation was followed by [12]. In [13], [16], [17] and [19] the translation coefficient equals 4.08×10^{-3}s^{-1}, while in [18] it equals 6.67 $\times 10^{-3}$s^{-1}.

As already said even unrealistically high transcription coefficients may be compensated by smaller translation coefficients, so the entire model can give correct predictions of proteins kinetics.

Protein and mRNA degradation coefficients

$c_3 = 7.5 \times 10^{-4}$s^{-1} – A20 and IκBα mRNA degradation rates. This value was re-fitted based on our pulse-pulse experiment, but remains in the range determined by Blatner et al. [2] who found that IκBα mRNA half life time is between 15 and 30 min.; $c_3 = 7.5 \times 10^{-4}$s^{-1} corresponds to 16 min. half live time. In [9] and [10], $c_3 = 4 \times 10^{-4}$s^{-1}. IκBα mRNA degradation rate in [13], [19] and [16] is equal 2.8×10^{-4}s^{-1}, in [17] it equals 5.6×10^{-4}s^{-1} and in [18] it equals 13.3×10^{-4}s^{-1}.

$c_5 = 5 \times 10^{-4}$s^{-1} – A20 degradation rate; this value was re-fitted mostly based on our pulse-pulse experiment. In previous works [9], [10], $c_5 = 3 \times 10^{-4}$s^{-1}

$tp = 0.01$ s^{-1} – degradation of P-IκBα and P-IκBα bounded to NF-κB. In all previous model the immediate degradation of phospho -IκBα was assumed. Here we add two separate fast equations for this process. The main reason is that the inhibition of proteasome can slow down degradation rate tp and that concentration of P-IκBα form can be measured, which potentially may help in model validation.

$c_{5a} = 10^{-4}$ s^{-1} – spontaneous degradation of IκBα, and $c_{6a} = 2 \times 10^{-5}$ s^{-1} spontaneous degradation of IκBα bounded to NF-κB, these values were adopted after Pando and Verma [5]. The same values are in [9], [13], [10] and [18].

Substantially different values are in recent works [16], [17] and [19], $c_{5a} = 2 \times 10^{-3}$s^{-1} and $c_{6a} = 10^{-6}$ s^{-1}. Specifically it was found in [19] that NF-κB binding slows down IκBα spontaneous degradation by a factor of 2000. Surprisingly they found that while IKK speeds up IκBα degradation when it is bounded to NF-κB, it slows down degradation of free IκBα. In our opinion these finding (especially the 6 min. half time for free IκBα protein) still deserve independent verification, since they seems to be in some contradiction with IκBα transfection experiments in which excess of IκBα is observed for hours. The other problem is that even basal IκBα transcription rate of 1.5 mRNA/s per gene copy appears to be above physiologically plausible level.

Transport coefficients

The transport characteristics of IκBα, NF-κB and IκBα|NF-κB complexes were examined by Carlotti et al. [6], who concluded that NF-κB nuclear import is 50 fold faster than export, while nuclear import of IκBα|NF-κB complexes is negligible. In other words they found that free NF-κB quickly translocates to the nucleus and its export back to the cytoplasm is due to its binding to

IκBα; IκBα|NF-κB complexes quickly migrate to cytoplasm. Based on IκBα overexpression studies Carlotti et al. [6] assumed that the ratio of IκBα transport parameters (nuclear import)/(nuclear export) =2. Hoffmann et al. [13], based on Carlotti et al. [6] and their model fits choose $i_{1a} = 3 \times 10^{-4}\text{s}^{-1}$, $e_{1a} = 2 \times 10^{-4}\text{s}^{-1}$, $i_1 = 0.09\text{s}^{-1}$, $e_{2a} = 0.0138\text{s}^{-1}$ (notation as in Table 1) and NF-κB nuclear export as $8 \times 10^{-5}\text{s}^{-1}$. With small modifications these parameters are then by followed subsequent works [16], [17], [18] and [19].

In this model we totally neglected the NF-κB nuclear export and fitted, NF-κB nuclear import, $i_1 = 0.01\text{s}^{-1}$, IκBα|NF-κB nuclear export: $e_{2a} = 0.05\text{s}^{-1}$, I$\kappa$B$\alpha$ nuclear import, $i_{1a} = 0.002\text{s}^{-1}$, and I$\kappa$B$\alpha$ nuclear export, $e_{1a} = 0.005\text{s}^{-1}$. The first two values are in basic agreement with Carlotti et al. [6] study, while the last two are not. Our coefficients $i_{1a} = 0.002\text{s}^{-1}$ and $e_{1a} = 0.005\text{s}^{-1}$ adopted here after [10] imply that free IκBα is rather cytoplasmic than nuclear. When fitting the model we realized that choosing $e_{1a} > i_{1a}$ we obtain more accurate fits. We expect that the source of this discrepancy is the following: Carlotti et al. [6] consider IκBα overexpressions for which the amount of IκBα is several fold higher than that of NF-κB. In real situation the excess of IκBα over NF-κB is not as significant. In fact, as shown by Yang et al. [22] (Fig. 4A) at low level of pIκB$\alpha-$EGFP transfection, IκBα, which is then expected to be in excess over NF-κB, is mostly cytoplasmic. The same can be observed in Nelson et al. [23] (Fig. 3A) experiment on Hela cells cotransfected with IκB$\alpha-$EGFP and RelA-DsRed-Express. Analyzing time series of images we may observe that for various ratios of IκB$\alpha-$EGFP:RelA-DsRed-Express, these two proteins remain in the cytoplasm. It appears that when IκBα is present in moderate excess over NF-κB, it remains mostly in cytoplasm. One could hypothesize that additional IκBα molecules may weakly associate with IκBα|NF-κB complexes, which would slow down nuclear import of these semi-free IκBα molecules. Experiment by Malek et al. [4] suggests that NF-κB heterodimers may have some additional IκBα binding sites.

It is not straightforward to compare transport coefficients of our model, with those of [13], [16], [17], [18], since in the Hoffmann/Levchenko models (except [12]) the "transport of concentrations" is considered, i.e. the nuclear and the cytoplasmic volume are implicitly assumed to be equal. This can imply that in fact the discrepancy between our models is smaller than it appears; for example IκBα concentration ratio (nucleus to cytoplasm) 2:1 implies inverse molecule number ratio 1:2.5 (assuming $k_v = V/U = 5$, as we did in our model).

Miscellaneous

NF-κB$_{tot} = 10^5$– the total number of NF-κB molecules, assumed. Carlotti et al., [1] estimated number of NF-κB molecules as 60,000. It can be, however, substantially higher in Rel A transfected cells, [22]. Here we adopted the value of 100,000 to be somewhere in between those of the normal and transfected cells. In our previous works we assumed [9], [10] NF-κB$_{tot}$ = 60,000, but in [10] we found that the best agreement with Nelson et al. [23] experiment is for NF-κB$_{tot}$ = 120,000. In the original Hoffmann et al. model [13], and in subsequent works [16], [18], [19] NF-κB concentration is assumed to be 100nM. In Kearns et al. [17], total concentration of NF-κB containing complexes is 0.125nM equal to 125,000 NF-κB molecules when calculated per cytoplasmic volume.

$a_1 = 5 \times 10^{-7}\text{s}^{-1}$ – IκBα–NFκB association (in cytoplasm), and $a_1 \times k_v$ in nucleus. This high value was adopted after our previous study [10]. In corresponds to 0.5 $\mu M^{-1}\text{s}^{-1}$ as assumed by Hoffmann et al. [13]. It is known [4] that IκBα and NFκB have affinity of the order of 1nM, which causes that free IκBα and NF-κB practically can not be observed together. For simplicity, we neglected dissociation of IκBα|NFκB complexes in the model.

References

[1] Carlotti F, Chapman R, Dower SK, Qwarnstrom EE: **Activation of Nuclear Factor kB in Single Living Cells.** *J Biol Chem* 1999, **274**:37941–37949.

[2] Blattner C, Kannouche P, Litfin M, Bender K, Rahmsdorf HJ, Angulo JF, Herrlich P: **UV-induced stabilization of c-fos and other short-lived mRNAs**. *Mol Cell Biol* 2000 **20**:3616–3625.

[3] Grell M, Wajant H, Zimmermann G, Scheurich P: **The type 1 receptor (CD120a) is the high-affinity receptor for soluble tumor necrosis factor.** *Proc Natl Acad Sci USA* 1998, **95**:570–575.

[4] Malek S, Huxford T, Ghosh G: **IκBα Functions through Direct Contacts with the Nuclear Localization Signals and the DNA Binding Sequences of NF-κB.** *J Biol Chem* 1998 **273**: 25427–25435.

[5] Pando MP, Verma IM: **Signal-dependent and -independent degradation of free and NF-κB-bound IκBα**. *J Biol Chem* 2000, **275**:21278–21286.

[6] Carlotti F, Dower SK, Qwarnstrom EE: **Dynamic Shuttling of Nuclear Factor kB between the Nucleus and Cytoplasm as a Consequence of Inhibitor Dissociation.** *J Biol Chem* 2000 **275**:41028–41034.

[7] Delhase M, Hayakawa M, Chen Y, Karin M: **Positive and negative regulation of IκB kinase activity through IKKβ subunit phosphorylation.** *Science* 1999, **284:**309-313.

[8] Park SG, Lee T, Kang HY, Park K, Cho K-H, Jung G: **The influence of the signal dynamics of activated form of IKK on NF-κB and anti-apoptotic gene expression: A systems biology approach.** *FEBS Letters* 2006, **580**:822-830.

[9] Lipniacki T, Paszek P, Brasier AR, Luxon B, Kimmel M: **Mathematical model of NF-κB regulatory module**. *J Theor Biol* 2004, **228**:195-215.

[10] Lipniacki T, Paszek P, Brasier AR, Luxon B, Kimmel M: **Stochastic regulation in early immune response**, *Biophys J* 2006, **90**:725-742.

[11] Levin, B., 2000. Genes, Vol. VII. Oxford University Press, Oxford.

[12] Cheong R, Bergmann A, Werner SL, Regal J, Hoffmann A, Levchenko A: **Transient IκB kinase activity mediates temporal NF-κB dynamics in response to wide rage of tumor necrosis factor-α doses**. *J Biol Chem* 2006, **281**:2945-2950.

[13] Hoffmann A, Levchenko A, Scott ML, Baltimore D: **The IκB - NF-κB signaling module: Temporal control and selective gene activation.** *Science* 2002, **298**:1241-1245.

[14] Alberts, B., et al., in Molecular Biology of the Cell. 2002, Garland: New York. p. 661.

[15] Swanson JA, Lee M, Knapp PE: **Cellular dimensions affecting the nucleocytoplasmic volume ratio.** *J Cell Biol* 1991 **115**:941-948.

[16] Werner SL, Barken D, Hoffmann A: **Stimulus specificity of gene expression programs determined by temporal control of IKK activity.** *Science* 2005, **309**:1857-1861.

[17] Kearns JD, Basak S, Werner ShL, Huang ChH, Hoffmann A: **IκBε provides negative feedback to control NF-κB oscillations, signaling dynamics, and inflammatory gene expression.** *J Cell Biol* 2006 DOI: 10.1083/jcb.200510155 JCB.

[18] Covert MW, Leung TH, Gaston JE, Baltimore D: **Achieving stability of Lipopolysaccharide-induced NF-κB activation**. *Science* 2005, **309**:1854-1857.

[19] O'Dea EL, Barken D, Peralta RQ, Tran KT, Werner ShL, Kearns JD, Levchenko A, Hoffmann A: **A homeostatic model of IκB metabolism to control constitutive NF-κB activity.** *Mol Sys Biol* 2007 **3** DOI:10.1038/msb4100148.

[20] Lee EG, Boone DL, Chai S, Libby SL, Chien M, et al.: **Failure to regulate TNF-induced NF-κB and cell death responses in A20-deficient mice.** *Science* 2000, **289**:2350-2354.

[21] Chan H, Aggarwall BB: **Role of tumor necrosis factor receptors in the activation of nuclear factor κB in human histiocytic lymphoma U-937 cells.** *J Biol Chem* 1994, **269**:31424-31429.

[22] Yang L, Ross K, Qwarnstrom EE: **RelA Control of IκB Phosphorylation.** *J Biol Chem 2003* **278**: 30881–30888.

[23] Nelson DE, Ihekwaba AEC, Elliot M, Johnson JR, Gibney CA, et al.: **Oscillations in NF-κB signaling control the dynamics of gene expression.** *Science* 2004, **306**:704-708.

[24] Nelson DE, Horton CA, See V, Johnson JR, Nelson G, et al.: **Response to comment on "Oscillations in NF-κB signaling control the dynamics of gene expression**. *Science* 2005, **308**:52b.

[25] Fujioka A, Terai K, Itoh RE, Aoki K, Nakamura T, Kuroda S, Nishida E, Matsuda M: **Dynamics of the Ras/ERK MAPK cascade as monitored by fluorescent probes.** *J Biol Chem* 2006 **281**:8917–8926.

[26] Femino AM, Fay FS, Fogarty K, Singer RH: **Visualization of single RNA transcripts in situ**. *Science* 1998 **280**:585–590.

[27] Bolouri H, Davidson EH: **Transcriptional regulatory cascades in development: initial rates, not steady state, determine network kinetics.** *Proc Natl Acad Sci USA* 2003 **100**:9371-9376.

[28] Bremer H, Yuan D: **RNA chain growth-rate in Escherichia coli.** *J Mol Biol* 1968 **38**:163-180.
